# Recent advances in nanoparticles targeting TGF-β signaling for cancer treatment

**DOI:** 10.7150/thno.126517

**Published:** 2026-01-01

**Authors:** Ziying Li, Xugang Ji, Xiaoyi Cong, Yu Gao, Jianqing Gao

**Affiliations:** 1 Key Laboratory of the Ministry of Education for Advanced Catalysis Materials, College of Chemistry and Materials Science, Zhejiang Normal University, Jinhua 321004, China.; 2 College of Pharmaceutical Sciences, Zhejiang University, Hangzhou 310058, China.; 3 Cancer Metastasis Alert and Prevention Center, College of Chemistry, and Fujian Provincial Key Laboratory of Cancer Metastasis Chemoprevention and Chemotherapy, Fuzhou University, Fuzhou 350108, China.

**Keywords:** nanodrug delivery system, TGF-β blockade, cancer treatment, nanoparticle, nanocarrier

## Abstract

Multiple therapies blocking TGF-β signaling have been investigated in preclinical and clinical trials over the past few decades; nevertheless, the outcomes of clinical trials are disappointing due to the double-faced systemic effects of TGF-β and the complexity of the tumor microenvironment. Intelligent nanodelivery systems engineered with responsive stimuli and targeting capabilities address the Janus-faced biology of TGF-β through spatially precise inhibition. Nanoparticles targeting TGF-β reciprocally create a positive feedback loop that enhances the penetration and delivery efficiency of nanoparticles because of the role of TGF-β in remodeling the tumor microenvironment. This review first outlines the function of TGF-β signaling, summarizes various tools for suppressing TGF-β signaling and provides an exhaustive emphasis on advanced nanoparticles targeting TGF-β. This review elucidates the symbiotic interplay between TGF-β blockade and nanoparticles, where nanomaterial-based strategies refine the specificity of TGF-β targeting, while the blockade of TGF-β reciprocally enhances the efficiency of nanoparticle-mediated delivery. Additionally, current challenges and future directions are highlighted to guide the future development of TGF-β blockade strategies and nanoparticles for antitumor therapy.

## 1. Introduction

Transforming growth factor-β (TGF-β) is a secreted cytokine that is involved in various biological activities, including tumor progression and metastasis, immunization inhibition and tissue reconstruction. TGF-β critically governs cellular proliferation, differentiation, apoptosis, and invasion across biological systems but presents a dualistic paradox in disease pathogenesis. During the initial phase of tumor development, it mediates tumor-suppressive effects through the induction of cell cycle arrest and apoptosis under normal physiological conditions 1. However, in advanced malignancies, TGF-β is transformed into an oncogenic driver, promoting the excessive deposition of extracellular matrix (ECM), angiogenesis, immunosuppression and epithelial-mesenchymal transition (EMT) 2. Although multiple factors increase the complexity of TGF-β signal transduction, numerous clinical trials have proven the favorable antitumor effect of TGF-β blockade 3,4. The most popular treatment strategy involves combining TGF-β pathway antagonists (such as antibodies, recombinant proteins, and small-molecule inhibitors) with other agents, including chemotherapeutic drugs 5, immune checkpoint inhibitors (ICIs, e.g., anti-PD-1/PD-L1) 6, and chimeric antigen receptor-modified (CAR)-T cells 7. However, systemic exposure, off-target effect, and poor tissue penetration of TGF-β pathway antagonists themselves restrict the clinical translation of TGF-β blockade 8.

The advent of nanotechnology has revolutionized strategies for the precise modulation of TGF-β, a Janus-faced cytokine implicated in fibrosis, cancer, and autoimmune diseases. These next-generation nanoplatforms not only potentiate the therapeutic index through pathological site-restricted action but also maintain the physiological homeostasis of TGF-β in nonneoplastic tissues, thereby resolving the conundrum of therapeutic specificity versus systemic preservation of its biological functions.

In other words, the TGF-β signaling pathway offers an innovative direction for designing nanoparticles (NPs) for combination therapy and increasing NP delivery efficiency. The effective delivery of NPs to tumor sites remains a tremendous challenge due to the complex path through which they must traverse the tumor microenvironment (TME), which also affects the therapeutic effect of NPs 9. TGF-β blockade could enhance delivery and penetration efficiency by weakening stromal barriers, improving vascular permeability and modulating immune responses. Pharmaceutical convergence between nanotechnology and TGF-β-targeted drug delivery systems (DDSs) has shown particular translational promise in multimodal therapeutic architectures that potentiate the effects of conventional chemotherapy, novel ICIs, and revolutionary immune cell therapy 10-12.

This review illuminates the symbiotic relationship evolving at the intersection of TGF-β biology and NPs, where nanotechnology-driven solutions increase the specificity of TGF-β targeting, whereas engineering of the TGF-β pathway reciprocally enhances the efficiency of nanotherapeutic delivery. Central to this discourse are NP systems capable of intrinsically neutralizing TGF-β via ligand scavenging or signaling cascade disruption, alongside advanced nanocarriers engineered for the spatiotemporally controlled delivery of TGF-β pathway antagonists such as monoclonal antibodies, gene-silencing RNAs, and TGF-β ligand traps. Concurrently, TGF-β inhibitors (TGF-βi) are being repurposed to potentiate nanodrug penetration by remodeling pathological microenvironments, such as by decompressing the fibrotic stroma or activating the immunosuppressive TME, thereby establishing a self-reinforcing therapeutic loop. Emerging frontiers further exploit multifunctional nanoplatforms that integrate TGF-β blockade with complementary modalities, including immunotherapies and conventional chemoradiation, capitalizing on the pleiotropic role of TGF-β in orchestrating multimodal antitumor responses. This bidirectional interplay represents a remarkable advancement intumor treatment, where nanotechnology and TGF-β targeting work together to specifically inhibit cancerous areas while protecting healthy tissues, effectively balancing their dual effects.

## 2. TGF-β signaling and function in tumor cells

TGF-β is a member of the secreted cytokine family, which is composed of three isoforms of TGF-β (TGF-β1, -β2, and -β3), activins, bone morphogenetic proteins (BMPs), and growth and differentiation factors (GDFs). In particular, TGF-β1 is the most comprehensively and actively researched family member. TGF-β receptors (TβRs) are high-affinity binding proteins located on the cell membrane and can be divided into three subtypes: TβR-I, TβR-II, and TβR-III. Almost all cells in the body can secrete TGF-β, whose receptor also exists on the cell surface 13.

The TGF-β signaling pathway plays an overarching role in diverse biological activities through the canonical suppressor of mothers against decapentaplegic (SMAD) -dependent pathway and non-SMAD pathways. TGF-β initiates key biological events primarily through the phosphorylation of SMAD2 and SMAD3. These transcription factors are pivotal SMAD family members and essential components of the TGF-β signaling cascade 14. Activated SMAD2 and SMAD3 interact with SMAD4 to form a heterotrimer and enter the nucleus to modulate transcriptional activity. Non-SMAD signaling involves the collective pathways and downstream cascades triggered by TGF-β, including phosphorylation, acetylation, sumoylation, ubiquitination, and protein-protein interactions. For instance, TGF-β is known to initiate the non-SMAD signal-regulated kinases RAS, MEK1/2, and ERK1/2 to complete signal transduction.

TGF-β acts as a tumor suppressor in normal and premalignant cells but undergoes a functional switch to become a tumor promoter in advanced malignancies. In the initial phases of tumor development, the canonical TGF-β/SMAD 4 signaling pathway plays a tumor-suppressive role by inducing cell cycle arrest and apoptosis 15. SMAD4 plays a critical role at the G1/S checkpoint by inducing cell cycle arrest in the G1 phase through several cyclin-dependent kinase (CDK) inhibitors, including p15, p21, and p27 16,17. TGF-β induces intrinsic and extrinsic apoptosis through the modulation of Bcl-2 family members, DAPK, caspases, FAS, and DNA damage-inducible (GADD) 45-β. TGF-β promotes apoptosis primarily through the SMAD-dependent pathway by upregulating proapoptotic genes (e.g., BIM, PUMA, and NOXA), but it can also induce apoptosis via non-SMAD pathways. These pathways include the activation of kinase signaling, such as JNK/p38 MAPK signaling, which in turn modulates the activity and expression of Bcl-2 family proteins 18. Furthermore, TGF-β signaling initiates key executors such as DAPK, activate the caspase cascade, increase FAS-mediated signaling, and induce the expression of DNA damage-responsive genes (e.g., GADD45β).

**Figure 1:**
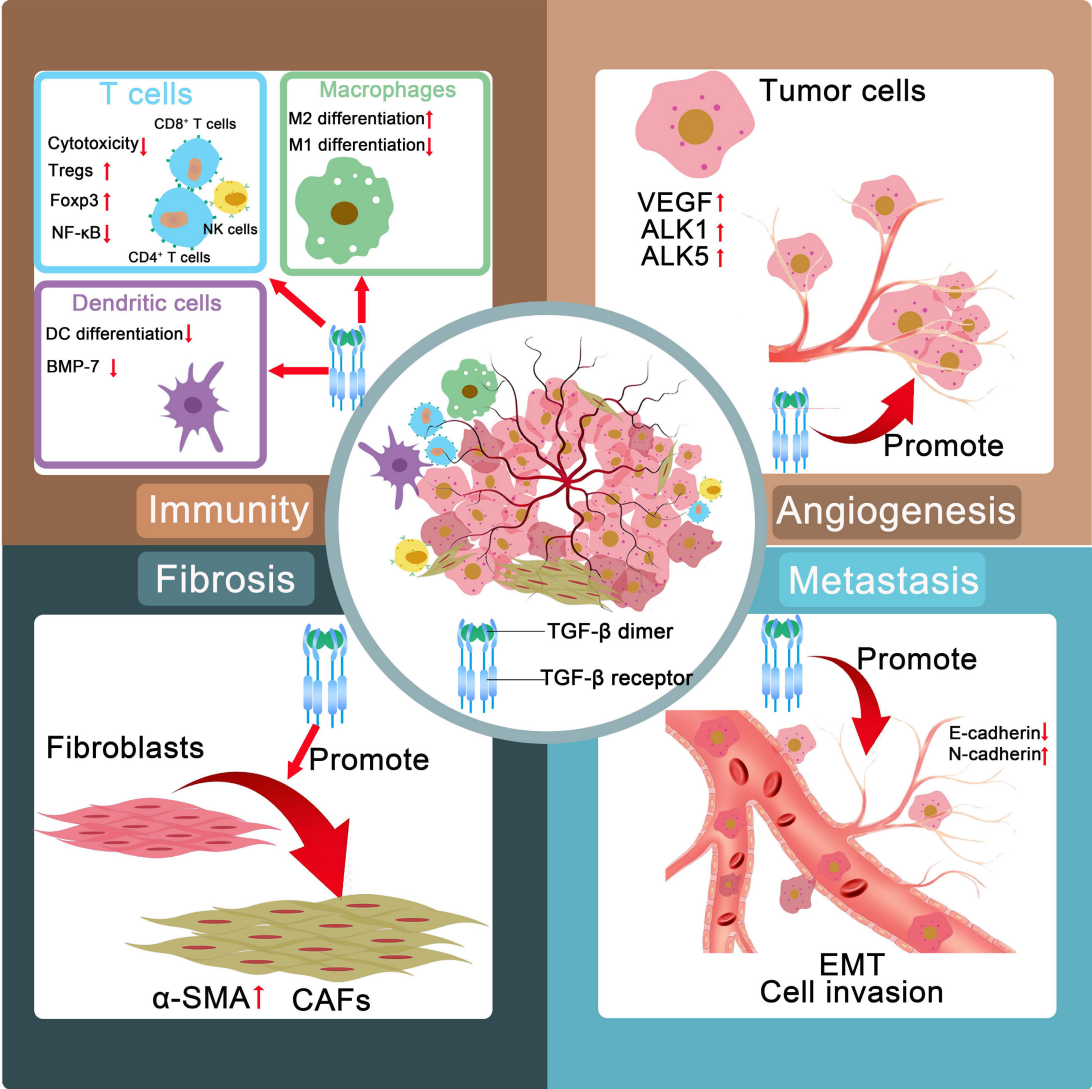
The role of TGF-β in late-stage tumors includes ECM construction, angiogenesis, immunosuppression, and the promotion of EMT. TGF-β: transforming growth factor-β; Tregs: regulatory T cells; Foxp3: forkhead box protein P3; NK cells: natural killer cells; BMP: bone morphogenetic protein; NF-κB: nuclear factor κB; DC: dendritic cell; CAFs: cancer-associated fibroblasts; α-SMA: α-smooth muscle actin; VEGF: vascular endothelial growth factor; ALK: anaplastic lymphoma kinase; EMT: epithelial-mesenchymal transition; ECM: extracellular matrix.

In advanced cancer stages, TGF-β facilitates ECM construction, angiogenesis, immunosuppression, EMT, and tumor aggression and metastasis (**Figure 1**). TGF-β orchestrates a protumorigenic microenvironment by driving ECM remodeling and fibrosis, leading to increased tissue stiffness and enhanced tumor invasion. TGF-β also promotes angiogenesis, ensuring a blood supply for the growing tumor. Crucially, it acts as a master regulator of immunosuppression by impairing the function of cytotoxic T cells and natural killer (NK) cells. Furthermore, TGF-β acts as a primary and crucial EMT inducer in late-stage tumors.

### 2.1 ECM construction

The TME consists of abnormal vasculature, a high density of tumor ECM and diverse cells (cancer-associated fibroblasts (CAFs), immune cells, endothelial cells, and so forth). The ECM is a dynamic supramolecular network that consists of collagens, fibronectin, elastin, glycosaminoglycans and other proteins and affects the progression and prognosis of tumors. CAFs, as vital facilitators of tumor growth and progression, are identified by the expression of α-smooth muscle actin (α-SMA), vimentin, and collagen I. The activation of the SMAD pathway, mediated via TβRII and TβRI, primarily drives the expression of proteins responsible for collagen deposition and matrix formation 19.

TGF-β induces the differentiation of fibroblasts into activated fibroblasts with increased expression of α-SMA. This marker is widely regarded as the dominant feature associated with CAFs 20. CAFs are derived from other resident stromal cells, such as epithelial or endothelial cells, through TGF-β-induced EMT. The differentiation of bone marrow-derived cells into CAFs is partially driven by TGF-β. And blockade of TGF-β can prevent mesenchymal stem cells from differentiating into CAFs 21.

### 2.2 Angiogenesis

The progression of solid tumors relies heavily on the establishment of functional tumor-associated blood vessels, which not only facilitate nutrient and oxygen delivery to proliferating neoplastic cells but also serve as conduits for metastatic dissemination. Notably, this intricate process of tumor vascularization requires coordinated ECM remodeling to provide structural support for the maturation of the nascent vasculature. Emerging evidence positions TGF-β as a pivotal orchestrator of this pathological process through multifaceted regulatory mechanisms.

TGF-β exerts dual regulatory effects on vascular homeostasis by differentially modulating the anaplastic lymphoma kinase (ALK1 and ALK5) signaling pathways, thereby balancing endothelial cell proliferation and stabilization while simultaneously increasing ECM protein deposition to increase vascular integrity. Furthermore, this pleiotropic cytokine demonstrates an immediate proangiogenic capacity by rapidly inducing vascular endothelial growth factor (VEGF) secretion, establishing a hypoxic TME that perpetuates angiogenic signaling cascades 22.

Beyond its immediate effects on endothelial activation, TGF-β coordinates comprehensive vascular maturation through a novel TGF-β-fibronectin signaling axis. This mechanotransduction pathway enhances pericyte-endothelial cell interactions, effectively stabilizing the immature vasculature while promoting the development of perfusion-competent blood vessels capable of sustaining tumor growth 23,24. This multidimensional regulatory capacity positions TGF-β as a crucial regulator of tumor angiogenesis, simultaneously governing matrix remodeling, growth factor signaling, and vascular structural organization through distinct yet synergistic molecular mechanisms.

### 2.3 Immunosuppression

TGF-β induces immunosuppressive effects through multiple cell types. In natural killer (NK) cells, it attenuates cytotoxic activity via the dual inhibition of IFN-γ synthesis (through STAT5 pathway blockade) and effector molecule expression (via SMAD3-mediated transcriptional suppression of perforin/granzyme B) 25. With respect to CD4^+^ T cells, TGF-β inhibits the differentiation of both Th1 and Th2 lymphocytes while simultaneously facilitating the generation and functional maturation of Th17 cells, Th9 cells, and regulatory T cells (Tregs). Notably, TGF-β is among the most powerful immunomodulatory cytokines involved in orchestrating Th1 cell differentiation and modulating its effector functions, demonstrating a dual regulatory capacity that positions it as a pivotal mediator of immune homeostasis 26. TGF-β is capable of inducing the differentiation of CD8^+^ T lymphocytes into forkhead box protein P3 (Foxp3)-expressing Tregs 27. TGF-β facilitates the phosphorylation of SMAD2 and Akt while concurrently inducing the formation of SMAD2/p-SMAD2 and Akt/p-Akt complexes, ultimately leading to the suppression of monocyte-derived dendritic cell (DC) differentiation. This molecular mechanism contributes significantly to tumorigenesis and malignant progression through the establishment of immunosuppressive microenvironments 28.

In addition, TGF-β drives the expansion and functional maturation of Tregs by transcriptionally activating Foxp3, thereby amplifying immune tolerance within the TME. Concurrently, TGF-β orchestrates macrophage polarization by suppressing nuclear factor κB (NF-κB)-mediated proinflammatory signaling, redirecting these myeloid cells toward a protumorigenic M2 phenotype characterized by arginase-1 overexpression and interleukin-10 secretion 29. Notably, the synergistic interplay between Treg-derived immunosuppressive cytokines and M2-polarized tumor-associated macrophage (TAM)-secreted trophic factors establishes a self-reinforcing immunosuppressive mechanism, effectively paralyzing cytotoxic T lymphocyte (CTL) activity while promoting tumor immune evasion. The dense ECM driven by TGF-β also restrains the deep penetration of immune cells besides the direct immunosuppressive effect of TGF-β 30.

### 2.4 Induction of EMT

During the EMT process, epithelial cells acquire mesenchymal characteristics, which enhances their migratory ability, invasiveness, and resistance to apoptosis 31. In cell culture, epithelial cells stimulated with TGF-β exhibited a loss of E-cadherin expression and an elevated level of N-cadherin expression, along with morphological changes 32,33. TGF-β is considered among the earliest EMT inducers and has been verified to serve as a crucial modulator of cancer cell progression and metastasis. The TGF-β signaling pathway is predominantly mediated by canonical pathways involving the transcription factors SMAD2, SMAD3, and SMAD4 and multiple noncanonical pathways. An elevated intratumoral concentration of TGF-β induces alterations in the EMT state of tumor cells, which also triggers significant reprogramming of the TME. The metabolism of carcinoma cells is profoundly affected by TGF-β, resulting in the establishment of a hypoxic environment within the TME 34. Certain carcinoma cells may enter a dormant state, which could serve as a reservoir of therapy-resistant cells, laying the foundation for tumor recurrence 35-41. Accumulating evidence has indicated that TGF-β induces epithelial tumor cells to acquire a mesenchymal phenotype characterized by increased invasiveness 42. Thus, high expression of TGF-β1 has been verified to correlate with increased metastasis.

## 3. Blockade of TGF-β in cancer

**Figure 2:**
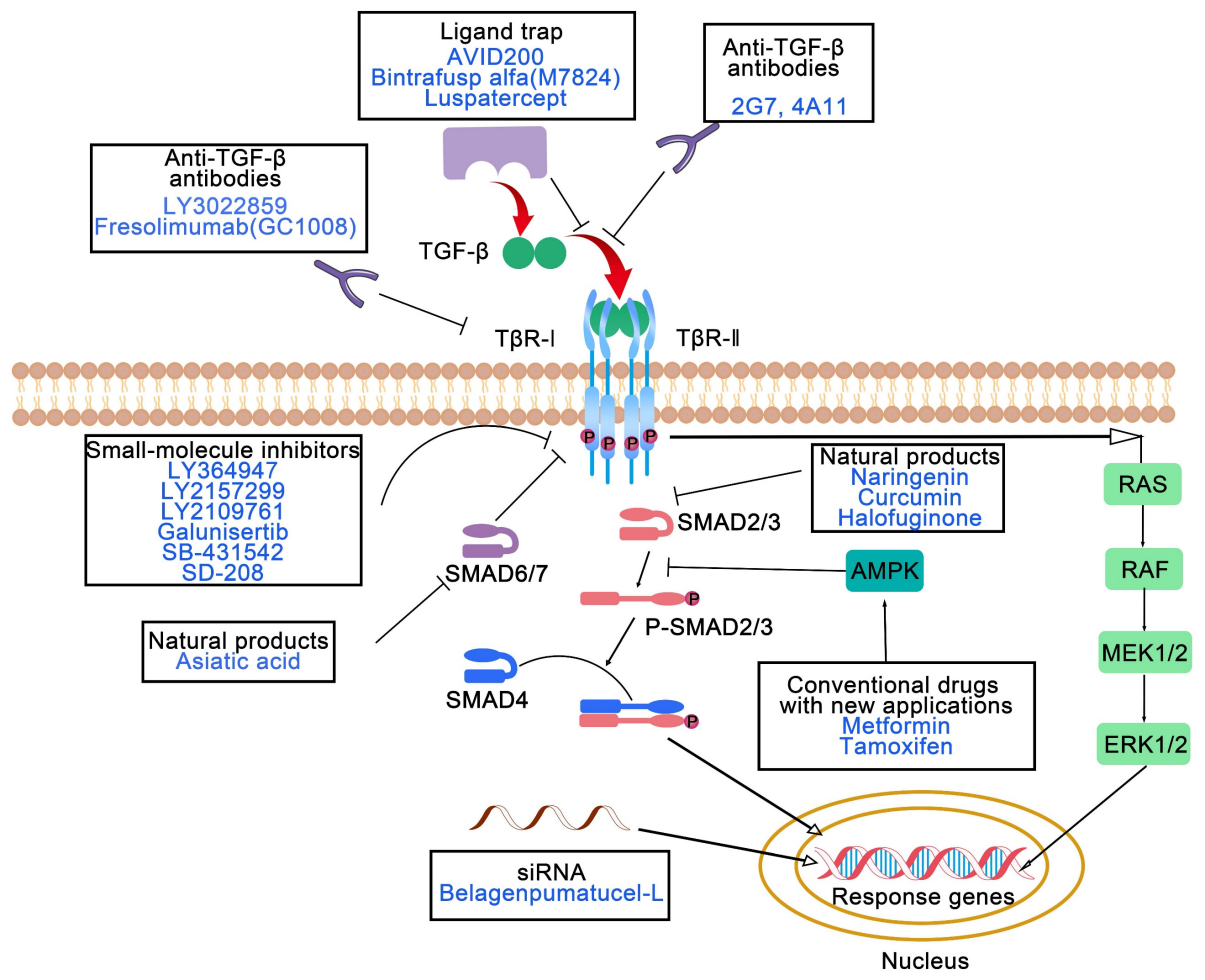
Molecular mechanisms of action of TGF-β pathway antagonists. TGF-β pathway antagonists can be classified into seven types: antibodies that inhibit ligand/receptor interactions, small-molecule inhibitors, antisense oligonucleotides, ligand traps, vaccines, natural products and conventional drugs with new applications. TGF-β: transforming growth factor-β; TβR: TGF-β receptor; SMAD: suppressor of mothers against decapentaplegic; AMPK: adenosine monophosphate-activated protein kinase; RAS: rat sarcoma; RAF: rapidly accelerated fibrosarcoma; MEK: mitogen-activated protein kinase kinase; ERK: extracellular signal-regulated kinases.

In terms of antitumor therapeutic targets, TGF-β pathway antagonists can be classified into seven types: antibodies that inhibit ligand/receptor interactions, small-molecule inhibitors, antisense oligonucleotides, ligand traps, vaccines, natural products and conventional drugs with new applications (**Figure 2**) 43. Numerous these agents have been or are being assessed in clinical trials against diverse tumors. The diverse agents are discussed in more detail below.

### 3.1 Antibodies that inhibit ligand/receptor interactions

Direct blockade of TGF-β ligand-receptor interactions using antibodies has been shown to be a highly specific and promising intervention. By inhibiting signal transduction at its source, these antibodies regulate the activity of the TGF-β pathway, providing therapeutic approaches for related diseases 44-46. 2G7 and 4A11 are two murine monoclonal antibodies that have been researched in drug-resistant animal models to restore sensitivity to cyclophosphamide (CTX) and cisplatin (CDDP) 47. The human monoclonal anti-TGF-β antibody LY3022859 suppresses the activation of TβR-I- and TβR-II-mediated signaling by preventing the formation of ligand-receptor complexes. However, a safe and effective dose could not be determined in the phase I study of LY3022859 because the TGF-β receptor is ubiquitously expressed in the body 48.

Another human monoclonal antibody, fresolimumab (GC1008), was broadly evaluated in clinical trials of patients with metastatic breast carcinoma 49, renal cell carcinoma 50 and other malignancies 51 to assess the clinical response. Compared with a lower dose of GC1008, a higher dose of GC1008 during radiotherapy was achievable and well tolerated, with efficient immune activation and a prolonged median overall survival among patients with metastatic breast cancer 49. Additionally, an analysis of functional T cells suggested that combining radiotherapy with GC1008 was ineffective at ameliorating T cell restriction by PD-1 52, which encouraged the strategy of the combination of dual PD-1 and TGF-β suppression and radiotherapy. In addition, numerous challenges remain in optimizing the affinity, specificity, and therapeutic effect of antibodies 53.

### 3.2 Small-molecule inhibitors

Numerous small molecules can be classified into three categories according to their structures: pyrazole, imidazole and pteridine compounds. Most TβR-Ⅰ inhibitors are pyrazole derivatives, such as LY364947 (HTS-466284), LY2157299 (galunisertib), and LY2109761. LY2109761 suppresses the TGF-β-induced liver metastasis and progression of pancreatic carcinoma through dual targeting of TβR-I/II activity 54. Galunisertib is being evaluated in clinical trials for the treatment of myelodysplastic syndrome, advanced hepatocellular carcinoma and metastatic pancreatic cancer. Data from phase II clinical trials (NCT01246986) have revealed that decreased TGF-β1 levels induced by galunisertib are related to longer overall survival in patients with advanced hepatocellular carcinoma 55. Galunisertib has advanced the most recent clinical trials among all small-molecule TGF-βi and has favorable pharmaceutical properties and selectivity. T LY2157299 was proven to facilitate the antitumor effect of nivolumab (anti-PD-1) in a phase II study (NCT02423343) 56.

TGF-βi based on imidazole and pteridine derivatives have been validated in preclinical studies but have not yet been tested in clinical trials. Both SB-431542 and its derivatives SB-505124 and SB-525334 possess an imidazole scaffold that suppresses the kinase activity of TβR-I, ALK4, ALK5 and ALK7. In preclinical models, SB-431542 has been demonstrated to have antitumor effects on breast carcinoma, colon cancer, osteosarcoma and malignant glioma 57-60. It abates immune suppression by mitigating the population of Treg cells and rescuing the proliferation of T cells in a murine fibrosarcoma model 61. The TβR-I kinase inhibitor SD-208 is the only molecule with a pteridine structure. According to a preclinical study in SMA-560 glioma-bearing syngeneic mice, SD-208 prolongs the survival by promoting immune responses 62. While small-molecule TGF-βi demonstrate remarkable therapeutic potential for improving pharmaceutical properties and synergistic efficacy in combination with ICIs, their clinical treatment remains limited by structural limitations that compromise target specificity and unresolved challenges in pharmacokinetic optimization across diverse TME. Despite the promising preclinical outcomes in terms of metastasis suppression and immune modulation, the long-term therapeutic efficacy of these compounds is unclear because of emerging compensatory signaling pathways and insufficient structural diversity within existing scaffolds to address heterogeneous tumor resistance mechanisms.

### 3.3 Antisense oligonucleotides

RNA interference (RNAi) is an innovative approach for the specific knockdown of gene expression. Only a few clinical trials in which TGF-β was silenced by a siRNA (siTGF-β) have been conducted for the treatment of non-small cell lung cancer (NSCLC) 63, advanced glioma 64, 65, and malignant melanoma 66. In a phase II study of belagenpumatucel-L 63, a TGF-β2 antisense gene-modified tumor cell vaccine, a dose-dependent difference in survival was observed, and no significant adverse events were detected among NSCLC patients. RNAi has notable clinical advantages because it involves the use of sequence-specific gene silencing mechanisms to precisely modulate the expression of TGF-β 67. Nevertheless, the clinical translation of RNAi therapeutics remains limited by intrinsic limitations, including the intrinsic instability of naked RNA molecules in the systemic circulation and inefficient tissue penetration of conventional delivery systems, which collectively contribute to reduced bioavailability and potential off-target effects 68. Functionalized nanoscale delivery platforms can substantially increase the pharmacokinetic stability of RNAi payloads while enabling ligand-directed tumor targeting through surface engineering modifications, thereby addressing current delivery challenges with increased spatial control over therapeutic agent distribution.

### 3.4 Ligand traps

In addition to traditional ligands or receptor inhibitors, the design of “ligand traps” capable of efficiently capturing TGF-β ligands has gained attention as a novel strategy for precise therapy 69. This strategy involves the engineering of receptor domains or fusion proteins that specifically bind to TGF-β ligands, thereby suppressing the downstream signaling pathways. Compared with traditional methods, ligand traps offer advantages such as high affinity, high specificity, and low toxicity, providing an original perspective and the potential to treat TGF-β-related diseases 70-73. Several TGF-β ligand traps, such as AVID200, bintrafusp alfa (M7824), and luspatercept, can target TGFβ ligands through fusion proteins and prevent ligand-receptor binding. M7824 is a bifunctional fusion protein and is composed of a TGF-β “trap” fused to anti-PD-L1 antibody. Bintrafusp alfa has been investigated in numerous preclinical and clinical trials for treatment of various tumors 74-81. Compared with monotherapy, dual suppression of PD-L1 and TGF-β by bintrafusp alfa results in effective antitumor effects in a preclinical animal model 80. A clinical trial (NCT04247282) has indicated that one or two neoadjuvant doses of bintrafusp alfa were well tolerated without adverse reactions or surgical delays 81. Dual PD-L1 and TGF-β inhibition efficiently enhanced T cell activation in a safe manner, increasing the feasibility of multimechanism neoadjuvant immunotherapy.

### 3.5 Vaccines

Vaccines targeting the TGF-β pathway, such as gemogenovatucel-T (Vigil, Vital) and belagenpumatucel-L (Lucanix), provide new emerging therapies with superior immunological effects in cancer treatment. Gemogenovatucel-T consists of a plasmid encoding the human immunostimulatory gene GMCSF and a bifunctional shRNA that specifically knocks down furin, TGF-β1 and TGF-β2 82. According to completed and ongoing clinical trials of gemogenovatucel-T or combination treatment with ICIs, the safety, efficient immune response, and antitumor effect of gemogenovatucel-T were confirmed in patients with different advanced gynecological cancers 83-88.

### 3.6 Natural products

Natural molecules derived from plants exhibit promising antitumor effects through the TGF-β signaling. Natural molecules suppressing TGF-β signaling include alkaloids, triterpenoids, diterpenoids, polyphenols, indoles, steroids, anthraquinone, and benzenoids. However, all these agents have been tested *in vitro* and *in vivo* but not in clinical trials. Triterpenoids, such as arjunolic acid 89, asiatic acid 90, betulinic acid 91, celastrol 92, and ginsenosides 93, 94, exhibit antitumor effects by suppressing TGF-β signaling. Lian *et al.* 90 reported that asiatic acid (a SMAD7 agonist) combined with naringenin (a SMAD3 inhibitor) synergistically regulated the TGF-β/SMAD signaling balance and inhibited the invasion and metastasis of melanoma and lung cancer. Diverse investigations have demonstrated that natural products effectively inhibit tumor invasion and metastasis by modulating the TME through the TGF-β signaling pathway. These studies provide experimental basis and potential clinical value of developing natural products that target TGF-β. For instance, curcumin not only suppressed EMT through TGF-β/SMAD2/3 signaling 95 but also reeducated CAFs by reducing α-SMA and COX-2 expression 96. Halofuginone, a plant alkaloid derivative, inhibited osteosarcoma progression against lung metastases 97 and melanoma against bone metastasis 98 by inhibiting TGF-β/SMAD3 signaling. Salvianolic acid B, obtained from *Salvia miltiorrhiza*, suppressed TGF-β1-induced EMT and is a promising therapeutic agent for treating NSCLC 99.

### 3.7 Conventional drugs with new applications

Increasing evidence has verified that some conventional drugs, such as metformin, tamoxifen and pirfenidone, exhibit anti-tumor effect through the regulation of TGF-β signaling. Metformin, a medicine applied to treat type 2 diabetes, suppressed the TGF-β1-induced EMT through the activation of adenosine monophosphate-activated protein kinase (AMPK) in various tumors, such as pancreatic carcinoma 100 and cervical carcinoma 101. Another study also revealed that metformin decreased the expression of ECM components by modulating PSCs and reeducating TAMs 102. Tamoxifen, an adjuvant chemotherapeutic, prolonged the survival of patients with almost all stages of estrogen receptor-positive breast carcinoma. Emerging evidence has indicated that the regulation of mitochondrial bioenergetics constitutes an innovative and efficacious approach to achieve the dual suppression of PD-L1 and TGF-β. Zhou *et al.* reported that tamoxifen efficiently suppressed PD-L1 and TGF-β expression by promoting AMPK phosphorylation 103. Pirfenidone, an FDA-approved drug for idiopathic pulmonary fibrosis, inhibits tumor metastasis by preventing TGF-β/SMAD signaling in triple-negative breast cancer (TNBC) 104 and reducing collagen and E-cadherin levels through the TGF-β signaling pathway in colorectal carcinoma 105.

## 4. Strategies for the design of nanodelivery systems targeting TGF-β in cancer therapy

Both TGF-βi and their co-administration with ICIs have been proven to exert therapeutic benefits in clinical trials. However, the clinical translation of TGF-βi such as galunisertib, fresolimumab, and bintrafusp alfa is still hampered by significant limitations, including rapid systemic clearance, off-target toxicity, and insufficient drug penetration. These challenges often result in unsatisfactory efficacy and harmful side effects, hindering successful clinical trial outcomes. Nanodelivery strategies involving the encapsulation of these therapeutics can enhance the precise delivery of TGF-βi with reduced side effects on normal tissues, providing an effective platform to overcome the shortcomings of TGF-βi. On the one hand, TGF-β therapeutic agents have improved the penetration and therapeutic efficacy of antitumor drug-loaded NPs; on the other hand, the emergence of nanodelivery carriers has provided an effective approach to guarantee the biosafety and attenuate the systemic toxicity of TGF-β pathway antagonists for TME modulation and cancer treatment.

Furthermore, NPs with engineered physicochemical properties exploit the enhanced permeability and retention (EPR) effect to achieve passive targeting and employ surface properties for active targeting, promoting specific accumulation and prolonged retention within the TME. This targeted aggregation significantly increases the local drug concentration where it is needed most, while simultaneously sparing normal organs. Moreover, researchers have increasingly shown that inorganic NPs may promote tumor metastasis through TGF-β signaling pathways, revealing the need for future investigations focusing on the potential adverse effects and safety concerns associated with NP applications. Consequently, these advanced strategies are pivotal for enhancing clinical trials for TGF-β blockade, enabling more informative and potentially successful evaluations of these promising agents in cancer treatment.

### 4.1 Sequential combination of nanoencapsulated TGF-β pathway antagonists and versatile NPs using a two-wave strategy

The convergence of nanotechnology and molecular targeting has catalyzed the development of multifunctional platforms capable of sequential microenvironmental normalization and tumor-specific payload delivery, exemplifying a new frontier in combinatorial therapeutic design. The following investigations adopted a sequential two-wave therapy: initial administration of nanoencapsulated TGF-β pathway antagonists to dismantle biological barriers within the TME through multifaceted mechanisms, followed by targeted delivery of drug-loaded NPs for cancer treatment. Sequential combination treatments involving TGF-β blockade and other therapeutic strategies, including chemotherapy, immunotherapy, and molecular targeted therapy, could allow for controlled spatiotemporal drug release and achieve efficient combination antitumor therapy (**Table 1**).

#### 4.1.1 Nanodelivery of TGF-β pathway antagonists to increase the efficacy of chemotherapy

**Figure 3:**
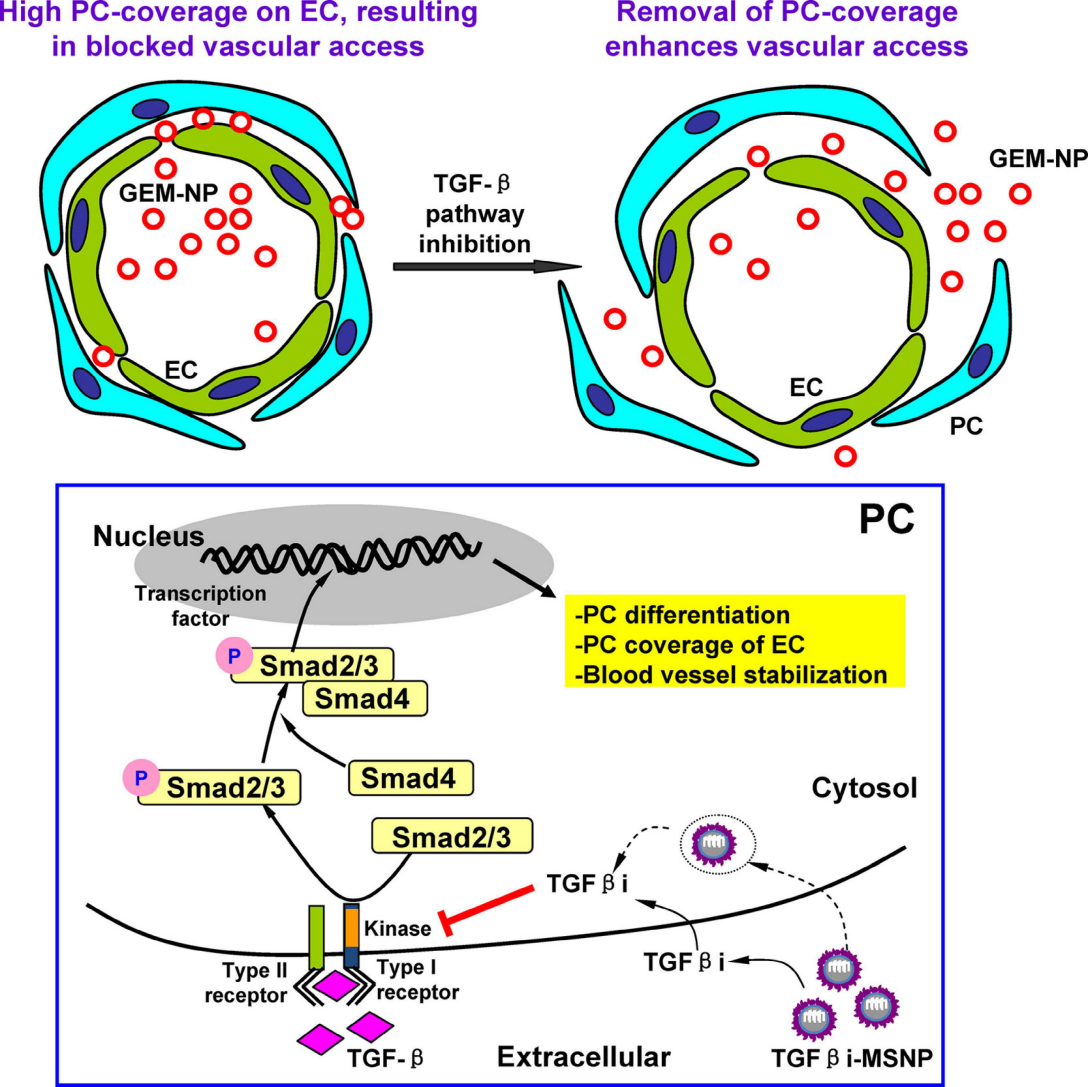
Schematic illustration of the two-wave strategy for TGF-β suppression and enhanced delivery efficiency of NPs. Adapted with permission from 106. Copyright 2013, American Chemical Society.

A challenging and central research objective is to improve the efficient delivery of nanomedicine, especially in pancreatic ductal adenocarcinoma (PDAC), in which the tumor comprises approximately 90% of the tumor stroma. The desmoplastic stroma of PDAC contains dense ECM and nontumor cells, especially pericytes. This stromal environment suppresses vascular fenestration and hinders the vascular access of NPs. Meng *et al.* demonstrated that polyethyleneimine (PEI)/polyethylene glycol (PEG)-coated mesoporous silica NPs (MSNPs) loaded with LY364947 efficiently reduced pericyte coverage of the vasculature by blocking TGF-β. Acting as a "first-wave" NPs, this treatment improved the accumulation of subsequent "second-wave" NPs (**Figure 3**) 106. PEI-PEG-encapsulated MSNPs were favorable for the secure delivery of inhibitors due to their ability to maintain monodispersity in biological fluids, as well as their prolonged circulatory half-life benefitting from the PEG coating. According to the results of the *in vivo* imaging studies, compared with single-wave MSNPs, LY364947-bound MSNPs clearly improved the accumulation and vascular access of “Hard” (PEI-PEG-coated MSNP) or “Soft” (liposome) NPs subsequently injected into BxPC3 xenografts. This sequential combination therapy improved the delivery efficiency of gemcitabine while reducing the systemic toxicity of the chemotherapy.

Another study revealed that the sequential delivery of the natural products α-mangostin and triptolide by two nanoplatforms represents a unique strategy for the treatment of desmoplastic PDAC 107. First, CAF-targeting CREKA peptide-modified PEG-PLA NPs (CRE-NP(α-M)) loaded with α-mangostin induced CAF deactivation, decreased collagen deposition, normalized the vascular system and improved blood perfusion by suppressing TGF-β/SMAD signaling 107. Subsequent tumor cell-targeting CRPPR peptide-modified pH-triggered micelles loaded with hydrophobic triptolide (CRP-MC(Trip)) exerted excellent tumor-suppressive effects on a PANC-1/NIH3T3 subcutaneous tumor model with negligible damage to normal organs 107. In addition to the most representative type of PDAC, chemotherapies for invasive breast cancer, melanoma and other desmoplastic tumors also face the problem of low penetration of chemotherapeutic drugs. The study and application of TGF-βi in other tumor models will offer novel strategies for the combined sequential treatment of desmoplastic tumors with superior therapeutic performance.

#### 4.1.2 Nanodelivery of TGF-β pathway antagonists to enhance the efficacy of molecular targeted therapy

The dense and desmoplastic stroma of PDAC is primarily mediated by TGF-β. Beyond this stromal component, KRAS mutation has been recognized as a pivotal oncogenic driver, accounting for nearly 90% of PDAC cases. TGF-β receptors mediate the phosphorylation of components of the KRAS pathway, whereas KRAS signaling regulates the phosphorylation of SMAD. The dual targeting of TGF-β and KRAS mutations may break through biological barriers and signal transmission for efficient treatment of PDAC. Pei *et al.* constructed CGKRK-conjugated NPs (Frax-NPCGKRK) for delivery of the antifibrotic agent fraxinellone to suppress TGF-β signaling and developed siRNA-loaded lipid-coated calcium phosphate (LCP) biomimetic NPs (siKras-LCPApoE3) to silence mutant KRAS, which led to the development of a novel sequential targeting strategy 108. NPs modified with TME-targeting peptide (CGKRK) could accurately deliver fraxinellone to CAFs in the TME, resulting in precise blockade of the TGF-β/SMAD pathway. Additionally, TGF-β blockade led to the inactivation of CAFs, a reduction in M2 macrophage polarization, and normalization of the vascular system in the TME, opening a new avenue for the subsequent delivery of siKras-LCP-ApoE3. This sequential strategy of TGF-β and silencing of mutant KRAS opens broader avenues for the treatment of PDAC.

#### 4.1.3 Nanodelivery of TGF-β pathway antagonists to facilitate immunotherapy

Cancer immunotherapy still faces challenges, such as limited immune responses against antigens and an immunosuppressive TME. Increased levels of immunosuppressive TGF-β might be responsible for poor immune responses. By reprogramming the TME, the Leaf Huang group constructed LPH NPs that delivered siTGF-β to improve the efficacy of immunotherapeutic LCP NP vaccines for advanced melanoma 109. A mixture of siTGF-β and hyaluronic acid condensed by protamine was coated with PEGylated liposomes through a stepwise self-assembly process to develop LPH NPs.

LCP NPs were designed with a calcium phosphate core for the codelivery of a mixture of the Trp2 (SVYDFFVWL) peptide and phosphorylated serine residues together with CpG oligonucleotides (ODNs), whose surfaces were modified with mannose to achieve increased accumulation in the lymph nodes. Considering that TGF-β plays a dual role in different stages of tumors, the authors started the TGF-β siRNA treatment on day 13 to guarantee that the tumors were in a later stage. LPH NPs loaded with siRNA led to approximately 50% knockdown of TGF-β expression, resulting in increased infiltration of CD8^+^ T cells and decreased levels of Tregs. Compared with the single-vaccine treatment, the combination of LCP NPs and LPH NPs facilitated 52% inhibition of tumor growth. Targeted silencing of TGF-β in advanced tumors resulted in a robust immune response to the vaccine and represents an original combination strategy against immunosuppressive tumors. More importantly, paying attention to the manifestations of the TGF-β inhibition strategy in early tumors will increase therapeutic safety and promote its clinical application.

In another study by the Leaf Huang group, a nanoemulsion (NE) formulation was developed using plutonic F68 and the targeting ligand DSPE-PEG-AEAA on the surface to deliver fraxinellone, which modulates the TME and facilitates the immunotherapeutic efficiency of mannose-modified lipid calcium phosphate NPs containing modified BRAFV600E peptide (pSpSSFGLANEKSI)) and a CpG oligodeoxynucleotide adjuvant in desmoplastic melanoma 110. In the absence of AEAA modification, compared with nontargeted NE, NE has a more preeminent tumor-targeting ability, indicating that fraxinellone inactivates CAFs. All these mechanistic insights collectively suggest that the spatiotemporal modulation of TGF-β signaling represents an innovative therapeutic approach for cancer management and is capable of simultaneously increasing treatment sensitivity and counteracting immune evasion.

#### 4.1.4 Nanodelivery of TGF-β pathway antagonists combined with vascular disrupting agents

As a promising class of antitumor drugs, vascular disrupting agents (VDAs) directly and precisely destroy tumor blood vessels, inducing secondary tumor necrosis and suppressing cancer progression through the provision of nutrients and oxygen to cancer cells. However, bleeding tissue contains large amounts of TGF-β1, which increases the risk of tumor metastasis. The combination of VDAs and TGF-βi could achieve powerful tumor suppression. Xu *et al.* selected methoxy-poly(ethylene glycol)-*b*-poly-(_D,L_-lactide) (mPEG_5k_-*b*-PLA_5k_) and maleimide-poly(ethylene glycol)-*b*-poly(_D,L_-lactide) (MalPEG_5k_-PLA_5k_) as carriers to encapsulate LY2157299 (LY) for the fabrication of A15-LY-NPs, whose surface was modified with coagulation-targeting peptide (A15) for accurate and efficient administration of TGF-βi to tumors 111. Considering the biphasic function of TGF-β in tumor progression, they first examined the effects of LY on early-stage tumors. Bioluminescent signals from *in vivo* optical imaging revealed that LY-NPs suppressed the establishment of 4T1-luc tumor cell colonies in the lungs and liver, exerting a tumor-suppressive effect on early-stage tumors. By employing a targeted nanoplatform for the precise delivery of TGF-βi, this promising treatment modulates the TGF-β pathway with fewer off-target effects. When further combined with a nanomedicine-loaded VDAs (CA4-NPs), a threefold increase in the intratumoral accumulation of A15-LY-NPs and a 93.7% inhibition of growth were observed in 4T1 tumors. This work presents a targeted strategy for the precise delivery of TGF-βi and a combination strategy for TGF-βi and VDAs.

### 4.2 Recent advances in versatile NPs blocking TGF-β for cancer treatment

**Figure 4:**
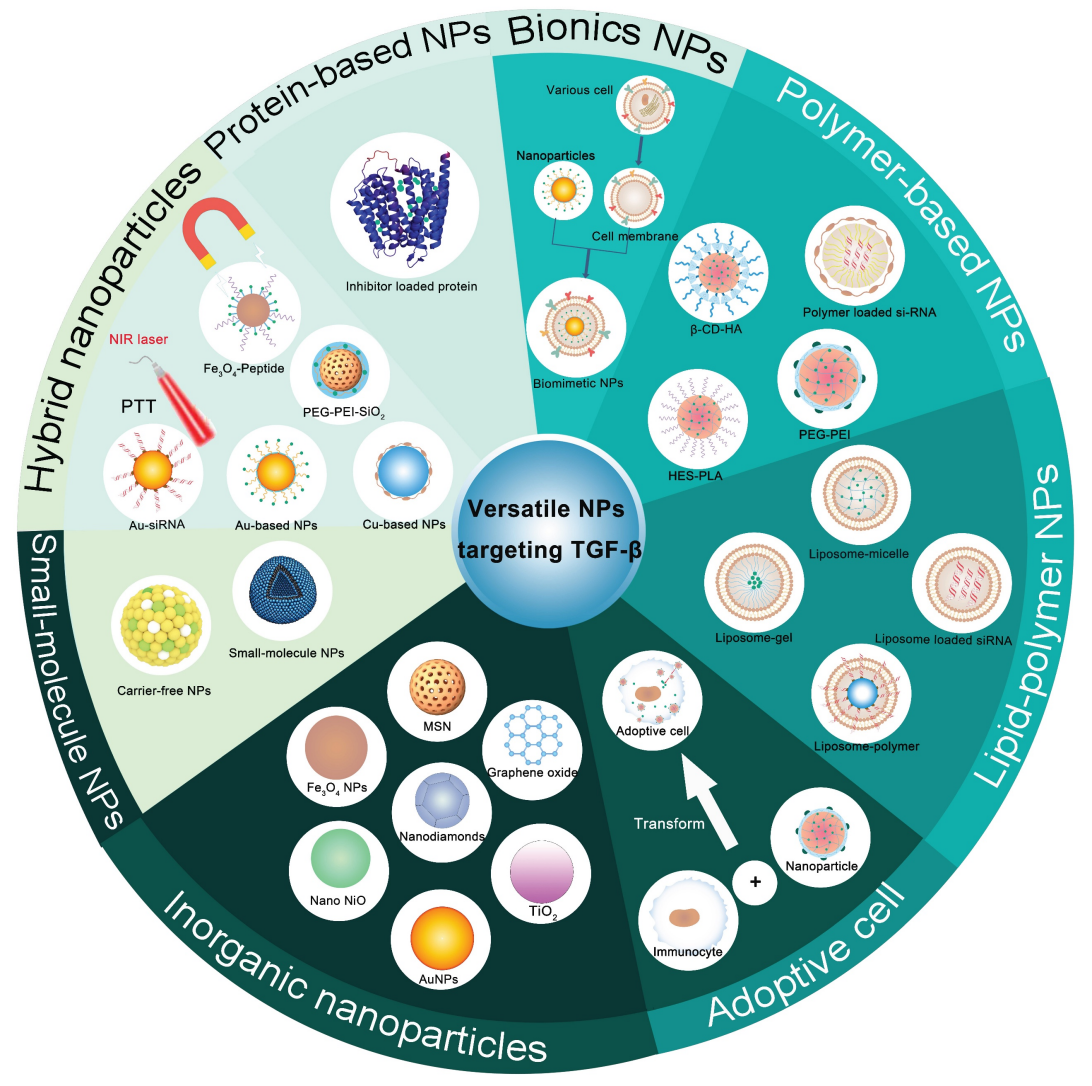
Schematic illustration of multiple NPs targeting TGF-β. NPs: nanoparticles; NIR: near-infrared; PTT: photothermal therapy; PEG: polyethylene glycol; PEI: polyethyleneimine; AuNPs: gold NPs; TiO_2_: titanium dioxide NPs; Nano NiO: nickel oxide NPs; MSN: mesoporous silica nanoparticle; HES: hydroxyethyl starch; PLA: polylactide; β-CD-HA: hyaluronic acid-aldehyde-monosubstituted β-cyclodextrin.

The majority of TGF-β therapeutic agents are associated with systemic toxicity and poor treatment outcomes. Targeted delivery of TGF-β therapeutic agents into the TME by versatile NPs (**Figure 4**) can not only achieve precise delivery of TGF-β pathway antagonists with enhanced therapeutic effects and safety but also open up a promising route for cancer treatment. Versatile NPs targeting TGF-β not only increase the spatial specificity and bioavailability of TGF-βi but also facilitate an improved intratumoral distribution of nanodrugs, thereby addressing key challenges in antitumor therapy, such as stromal barriers and heterogeneous drug delivery. The combination of stimulus-responsive release and stromal modulation represents a promising direction for the next generation of smart nanomedicines, potentially offering new avenues for improving therapeutic outcomes in solid tumors.

#### 4.2.1 Polymer-based NPs

Polymeric NPs with high drug loading capacity and modifiability have emerged as promising carriers to increase the solubility, stability, and targeted delivery of therapeutics, ranging from hydrophobic small molecules and genetic drugs. In addition, responsive polymeric NPs can respond to specific stimuli, releasing their payload in a spatiotemporally controlled manner upon exposure to internal (e.g., pH, enzymes, and redox) or external (e.g., light, temperature, and magnetic fields) stimuli; their tunable properties make them ideal for designing versatile NPs for TGF-β blockade and tumor treatment (**Table 2**).

##### 4.2.1.1 Enzymatic stimuli

Enzyme-responsive NPs can selectively react with specific enzymes expressed in tumor tissues, contributing to the precise release of therapeutics while minimizing side effects and improving therapeutic effects. Their adaptability makes them promising strategies for personalized and controlled therapy. For the codelivery of a hydrophobic TGF-βi and chemotherapeutic agents into the tumor site, hydroxyethyl starch-polylactide (HES-PLA) core-shell NPs (DOX/LY@HES-PLA) were established with dual α-amylase/pH-responsive properties to codeliver doxorubicin (DOX) and LY2157299 through ultrasonic emulsification and high-pressure homogenization 114. The hydrophilic shell formed by HES could be disintegrated by endogenous α-amylase, eliminating the detrimental “PEG dilemma” of the hydrophilic layer as a hopeful replacement for PEG and triggering the release of DOX or LY2157299. Compared with the combination of free inhibitor and free DOX, this codelivery nanosystem suppressed tumor growth and inhibited pulmonary metastasis more effectively. The heterogeneous drug distribution of chemotherapeutic drugs is considered a crucial factor contributing to the insufficient efficacy of chemotherapy, which, in turn, promotes EMT and metastasis through the activation of the TGF-β signaling pathway. For instance, DOX, cisplatin, paclitaxel and camptothecin upregulate TGF-β1 expression in various tumor cells. The inhibition of TGF-β could suppress tumor progression and the inadequate efficacy of chemotherapy caused by inhomogeneous drug distribution.

The TME of PDAC is characterized by a dense and desmoplastic stroma that is largely regulated by TGF-β, which promotes cancer progression, therapeutic resistance, and poor prognosis 115,116. Recent advances in nanotechnology and stroma-targeting strategies, particularly those designed to modulate TGF-β activity, have provided innovative approaches to overcome these challenges 117. Based on the high expression of matrix metalloproteinase-2 (MMP-2) by stromal cells and the overexpression of plectin-1 on the surface of pancreatic cancer cells, Li *et al.* constructed a MMP-2-responsive PPC nanopolyplex to deliver hydrophobic CPI-613 (an antimitochondrial metabolism agent), which self-assembled with the amphiphilic DSPE-PEG-plectin-1 peptide to form a plectin-1-targeting p-PPCL nanopolyplex to deliver the hydrophobic protein LY2109761 118. The tumor-responsive nanopolyplex was cleaved by MMP-2 in TEM, triggering the decomposition of the nanoplatform and the release of LY2109761. This “flower-like” nanopolyplex achieved stromal normalization through TGF-β blockade, which increased the accumulation and penetration of the nanopolyplex and enhanced the antitumor effect of free CPI-613 118. In normal, healthy tissues, the TGF-β signaling pathway is subject to stringent regulation. TGF-β ligands are synthesized and secreted in an inactive form, remaining latent within the extracellular space. Subsequent activation of these ligands can be mediated by proteolytic cleavage by enzymes such as MMP2 and MMP9. High expression of MMP-2 in tumor tissue is always accompanied by active TGF-β ligands. MMP-2-responsive NPs provide a directional nanoplatform for the accurate delivery of TGF-β pathway antagonists.

**Figure 5:**
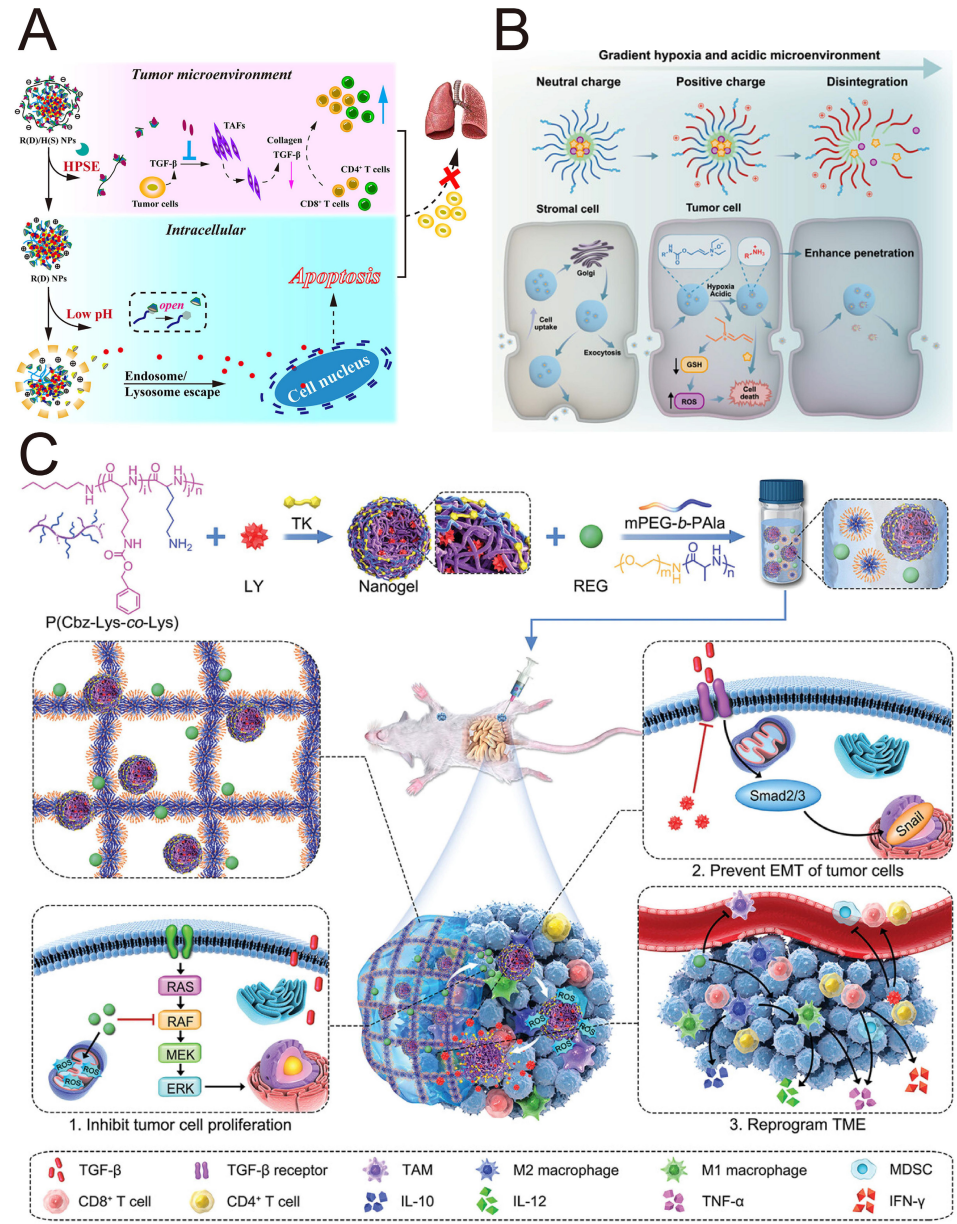
Studies of the polymer-based NPs targeting TGF-β for cancer treatment. (A) Schematic illustration of heparanase/pH-responsive NPs for TME modulation. Adapted with permission from 119. Copyright 2021, American Chemical Society. (B) Schematic illustration of hypoxia/pH-responsive charge-reversal NPs for deep penetration. Adapted with permission from 127. Copyright 2025, American Chemical Society. (C) Schematic illustration of a hydrogel/nanogel composite for sequential release of drug and synergistic therapy. Adapted with permission from 128. Copyright 2022, John Wiley and Sons.

The use of grafted polymer nanocarriers to load chemotherapeutic drugs and TGF-βi may amplify the chemotherapeutic effect and suppress tumor metastasis. Zhang *et al.* 119 constructed hierarchically releasing heparanase/pH-responsive NPs (R(D)/H(S) NPs) based on β-cyclodextrin-conjugated heparin and a pH-responsive pseudorotaxane to encapsulate DOX and SB431542 (**Figure 5A**). The negative potential of biodegradable heparin prevented SB431542 from being cleared by immune cells, but it could be degraded by heparinase in the TME, triggering the release of SB431542. This study revealed that R(D)/H(S) NPs inactivated CAFs by SB431542, which reduced the expression of collagen I, thereby remodeling the TME and subsequently enhancing the anti-tumor effect of DOX. Enzyme-responsive nanocomplexes protected TGF-βi from being cleared by immune cells and facilitated their entry into cells through lysosomal escape to exert their functions.

##### 4.2.1.2 Reactive oxygen species stimuli

Notably, reactive oxygen species (ROS), a crucial element of PDT, function as key mediators in the activation of latent TGF-β1 in the TME, ultimately causing its downstream detrimental signaling and sacrificing the therapeutic outcome of PDT. Han *et al.* revealed that PDT led to TGF-β1 accumulation-mediated immunosuppression through increased ROS/TGF-β1/MMP-9 and CD44-mediated local signaling 120. To opportunely block TGF-β once PDT is performed, they designed core-shell NPs (LC@HCDFC NPs) for the delivery of LY2109761 and chlorin e6 (Ce6, photosensitizer) to complement PDT by blocking TGF-β through the remodeling of immunosuppression 121. The amphiphilic shells were derived from a conjugate consisting of hyaluronic acid (HA)-aldehyde-monosubstituted β-cyclodextrin (HCD) and hexadecanol-conjugated ferrocene (FC), while the hydrophobic cores were composed of benzyl-modified poly(γ-glutamic acid) (BzPGA)-encapsulated LY210976 and Ce6. HCD conferred exceptional hydrophilicity but also increased CD44 receptor targeting efficiency via HA. Furthermore, the HCDFC inclusion complex facilitated the ROS-responsive release of LY210976, achieving timely inhibition of TGF-β once PDT was administered.

To cope with the immunosuppressive TME and address the low delivery efficiency of NPs, Dai *et al.* designed a pH/ROS-responsive size-loss and charge-reversal HCPT prodrug nanoplatform to condense siTGF-β for chemoimmunotherapy by reprogramming the immunosuppressive TME 122. This study leverages a symbiotic interplay between TGF-β inhibition strategies and DDSs. A size-tuneable, ROS-responsive nanoplatform enables the efficient and precise delivery of TGF-βi. Concurrently, the inhibition of TGF-β promotes the deep penetration of nanotherapeutic agents by modulating the TME.

##### 4.2.1.3 pH stimuli

Given the weakly acidic characteristics inherent to the TME, the rational design and engineering of pH-sensitive polymeric NPs emerges as a compelling strategic approach. In addition, electrostatic interactions drive the cellular uptake of positively charged NPs by negatively charged cancer cells. In a groundbreaking study, Huang *et al.* prepared a pH-responsive positively charged nanodrug via the self-assembly of poly(ethylene glycol)-block-poly(2-diisopropyl methacrylate) diblock copolymer (PEG-PDPA) using a nanoprecipitation method for the codelivery of the sonosensitizer Chlorin e6 (Ce6) and LY2157299 123. These positively charged NPs with much smaller particle sizes were conducive to efficient drug accumulation and endocytosis. This platform harnesses SDT to generate cytotoxic ROS bursts and induce robust immunogenic cell death (ICD), which simultaneously releases LY2157299 for the blockade of TGF-β/SMAD signaling and remodeling of the immune microenvironment. This strategy led to a 3.2-fold increase in anti-PD-L1 therapeutic efficacy against colorectal cancer liver metastases in preclinical models 123. In this study, SDT-mediated ICD, reversion of the immunosuppressive TME by TGF-βi and immune checkpoint blockade constitute an immune cycle for the treatment of immunologically cold tumors.

Hydrophobic polymeric materials with positive charge can be loaded with two functionally distinct drugs via layer-by-layer self-assembly and subsequently delivered to distinct cellular targets within the tumor region. Wang *et al.* developed pH-sensitive polymeric clustered NPs self-assembled by poly(ethylene glycol)-b-poly(ε-caprolactone) (PEG-PCL), a PCL homopolymer and poly(amidoamine)-grafted polycaprolactone (PCL-CDM-PAMAM) to codeliver LY2157299 and a siRNA targeting the PD-L1 gene for enhanced immunotherapy 124. Under conditions of acidic tumor extracellular pH, siPD-L1, which is attached to the surface of clustered NPs, is released with PAMAM as size-loss NPs to penetrate deep into the tumor tissue. LY2157299, which is loaded in the core of clustered NPs, exhibited sustained retention within the ECM to target pancreatic stellate cells (PSCs) because of the large size of the NPs. This nanoplatform enables the spatially and temporally controlled release of therapeutic agents, thereby synergistically cosuppressing the TGF-β pathway and the PD-L1 checkpoint for improved antitumor immunotherapy.

The encapsulation of TGF-βi into NPs could overcome the pH instability and hydrophobicity of TGF-βi, thereby facilitating its clinical translation. Yu *et al.* reported a new TGF-βi, the natural product ingenol-3-mebutate (I3A), which reduced the proportion of Tregs and IL-6 expression and promoted the intratumoral infiltration of CTL 125. After being encapsulated in polymeric vesicles with 'acidic nuclei' supplied by a single alcoholic hydroxyl (-CH(CH_3_)-OH), the obtained I3A-PM overcame the hydrophobicity and pH instability of I3A 125. The development of multiresponsive DDSs will offer a more robust platform for the targeted delivery of these plant-derived hydrophobic drugs.

The development of NPs capable of pH-responsive release under alkaline conditions is equally essential for preventing the degradation of TGF-βi in the acidic gastrointestinal environment. Yu *et al.* developed an elastic nanocarrier to release LY2157299 in the alkaline pH of the colon through the conjugation of polygalacturonic acid (PgA) and polyacrylic acid (PAA) while resisting the acidic pH of the gastrointestinal tract 126. These nanomicelles composed of PgA-PAA conjugates exhibited pH-responsive swelling behavior under alkaline conditions, thereby serving as effective stabilizers against the alkaline pH of the colon. Conversely, the aqueous solubility of PAA is limited at low pH, while its inherent mechanical elasticity provides structural support for the formation of nanomicelles, ensuring stability in the acidic pH of the gastrointestinal tract.

##### 4.2.1.4 Hypoxic stimuli

The dense ECM mediated by the TGF-β/SMAD signaling pathway causes the collapse of the vascular system, indirectly leading to the formation of a hypoxic acidic and immunosuppressive TME in PDAC. The tunable physical properties of the NPs (e.g., particle size and surface charge) allow for deep penetration and efficient regulation of the TGF-β pathway, making them a powerful nanosystem for TGF-βi delivery. Hypoxia/pH-responsive charge-reversal NPs (GemC18/Gal@CPLNO) with transcytosis functions were constructed by Zhang *et al.* to adapt to the pathological characteristics of PDAC (**Figure 5B**) 127. An amphiphilic polymer with hypoxia-responsive enamine N-oxides and the collagen-binding peptide CBP was synthesized to incorporate LY2157299 and a stearic acid-modified gemcitabine prodrug (GemC18) by self-assembly for stromal reprogramming and immune activation. Enamine N-oxides first achieved hypoxia-responsive transcytosis through self-sacrificial reduction to produce positively charged NPs. Simultaneously, the lysine residues of the polymer triggered cascade transcytosis by turning the neutral charge to a positive charge in response to the acidic TME. These positively charged NPs can initiate transcytosis across the ECM barrier to achieve deep penetration, releasing TGF-βi and other drugs in response to hypoxic and acidic microenvironments.

##### 4.2.1.5 Polymer-based hydrogels and nanogels

Hydrogels and nanogels represent two classes of biomaterials that are extensively utilized for controlled drug delivery. By incorporating nanogels into the three-dimensional network of hydrogels, the hydrogel/nanogel system achieves not only localized drug delivery through hydrogels whose mechanical properties match those of biological tissues but also cascade drug release by nanogels whose core can encapsulate another therapeutic agent. Li *et al.* constructed a thermosensitive hydrogel (Gel/(REG+NG/LY)) incorporating regorafenib and a ROS-responsive nanogel loaded with LY3200882 for spatiotemporally and sequentially controlled release in colorectal tumors (**Figure 5C**) 128. Owing to the prestumoral injection, this injectable hydrogel has fewer systemic side effects. The earlier thermosensitive release of REG increased the production of intracellular ROS after injection, splitting the thioketal linker within the nanogel and leading to the subsequent release of LY3200882. This sequential drug delivery nanosystem not only restrained the potential tumor metastatic threats induced by REG but also increased immune responses to the immunosuppressive TME through TGF-β blockade. This integrated hydrogel/nanogel system enables sequential and cascade drug release, thereby improving the spatiotemporal control of drug delivery and potentially increasing treatment efficacy in complex disease environments such as tumors.

##### 4.2.1.6 Gene medicine-loaded polymeric NPs

Polymeric carriers offer a great opportunity to package hydrophobic TGF-β and negatively charged gene medicines into one system for effective treatment. As TGF-β and IL-12 play diametrically opposing roles within the TME, coupling IL-12 delivery with TGF-β blockade could increase local immune responses to tumors, constituting a particularly potent treatment modality. Jiang *et al.* designed a versatile polymeric carrier, β-cyclodextrin-PEI, to encapsulate SB-505124 and the gene encoding murine IL-12 for immunotherapy of metastatic malignant melanoma 129. This nanosystem facilitated the sustained release of SB-505124 while ensuring efficient transduction of the adenoviral vector encoding the IL-12 gene. By incorporating responsive bonds into nanomaterials, responsive sequential release can be achieved for more precise combination treatments.

Multilayered polymeric NPs serve as excellent nanosystems to protect siRNAs targeting TGF-β1 (siTGF-β1) from inchoate decomposition and achieve the sustained release of gene drugs *in vivo*. Peres *et al.* constructed a poly(lactic acid) (PLA)-based nanovaccine for the codelivery of α-lactalbumin antigens, Toll-like receptor ligands, and glutamate chitosan/siTGF-β 130. This nanovaccine combined with the agonist immune checkpoint OX40 synergistically inhibited tumor progression and prolonged overall survival due to antigen-specific adaptive immunity and TGF-β silencing derived from the nanovaccine. This study further confirms that TGF-βi also improve the effectiveness of antigen-presenting cell checkpoint inhibitors, thereby broadening the scope of application of TGF-βi and providing valuable insights for clinical trials involving both ICIs and TGF-βi.

##### 4.2.1.7 Actively targeted polymeric NPs

Polymeric NPs engineered for active targeting can faithfully replicate the protracted growth dynamics of human tumors. T cell targeting NPs that accurately deliver immunomodulatory TGF-βi to tumor sites can improve T cell activity better than free TGF-βi and passive NPs can, with minor side effects. CD8^+^ T cells targeted polymeric NPs promote the accumulation of the immunomodulatory agent SD-208, restoring the activity of CTL and inhibiting tumor growth. Notably, the treatment achieves these effects at one logarithm lower dosage of anti-PD-1 and anti-SD-208, whereas free small-molecule drugs have no effects at the same dosage 131. Polymer-based NPs represent among the commonly employed drug delivery systems; however, strategies utilizing actively targeted polymeric systems for the delivery of TGF-βi remain relatively scarce. Further study is warranted to elucidate the relationship between TGF-β and TME-specific overexpression of receptors for the development of actively targeted DDSs.

#### 4.2.2 Inorganic NPs

Over the past few decades, inorganic NPs have presented a remarkable opportunity as drug delivery carriers because of their facile modification, stimuli-responsive drug release mechanisms, and imaging capability. Notably, inorganic NPs can intrinsically modulate the TGF-β signaling pathway, resulting in an antitumor effect through the blockade of TGF-β signaling in cancer or adverse effects on health through the activation of TGF-β (**Table 3**) 134. A focus on the biosafety of inorganic NPs and their side effects on normal tissues is crucial for promoting their clinical application.

##### 4.2.2.1 Inorganic NPs suppress the TGF-β signaling pathway

By serving as nanocarriers or therapeutic agents, gold NPs (AuNPs) are broadly applied because of their unique physical properties 135. AuNPs attenuate the EMT through the formation of AuNP-TGF-β1 conjugates, which result in the inactivation of the TGF-β1 136. Additionally, *in vivo* experiments indicated that AuNPs increased tumor immunity by increasing the infiltration of CD4^+^ and CD8^+^ T cells and interfering with TGF-β signaling in MBT-2 tumors 136. Zhang *et al.* demonstrated that AuNPs with a size of 20 nm inactivated CAFs by regulating the expression of fibroblast activation- or inactivation-associated markers, such as the blockade of TGF-β1, PDGF, and other markers 137. This study helps us determine the role of AuNPs in interfering with multicellular communication within the TME.

Carbon nanodiamonds, a unique class of carbon NPs, bind to TβR-II, promote its lysosomal degradation and inhibit the transduction of TGF-β signaling 138. Nanodiamonds suppress tumor invasiveness and induce macrophage polarization toward the antitumor M1 phenotype, indicating potential therapeutic effects on cancer. Metallofullerenol-based Gd@C_82_(OH)_22_ NPs, which are virus-like morphological nanocages, exert inherent antitumor effects on triple-negative breast cancer cells, with negligible side effects on normal mammary epithelial cells 139. Under normoxic and hypoxic conditions, the Gd@C_82_(OH)_22_ NPs eliminate CSCs and impede EMT by suppressing the expression of HIF-1α and TGF-β, further emphasizing their therapeutic potential.

**Figure 6:**
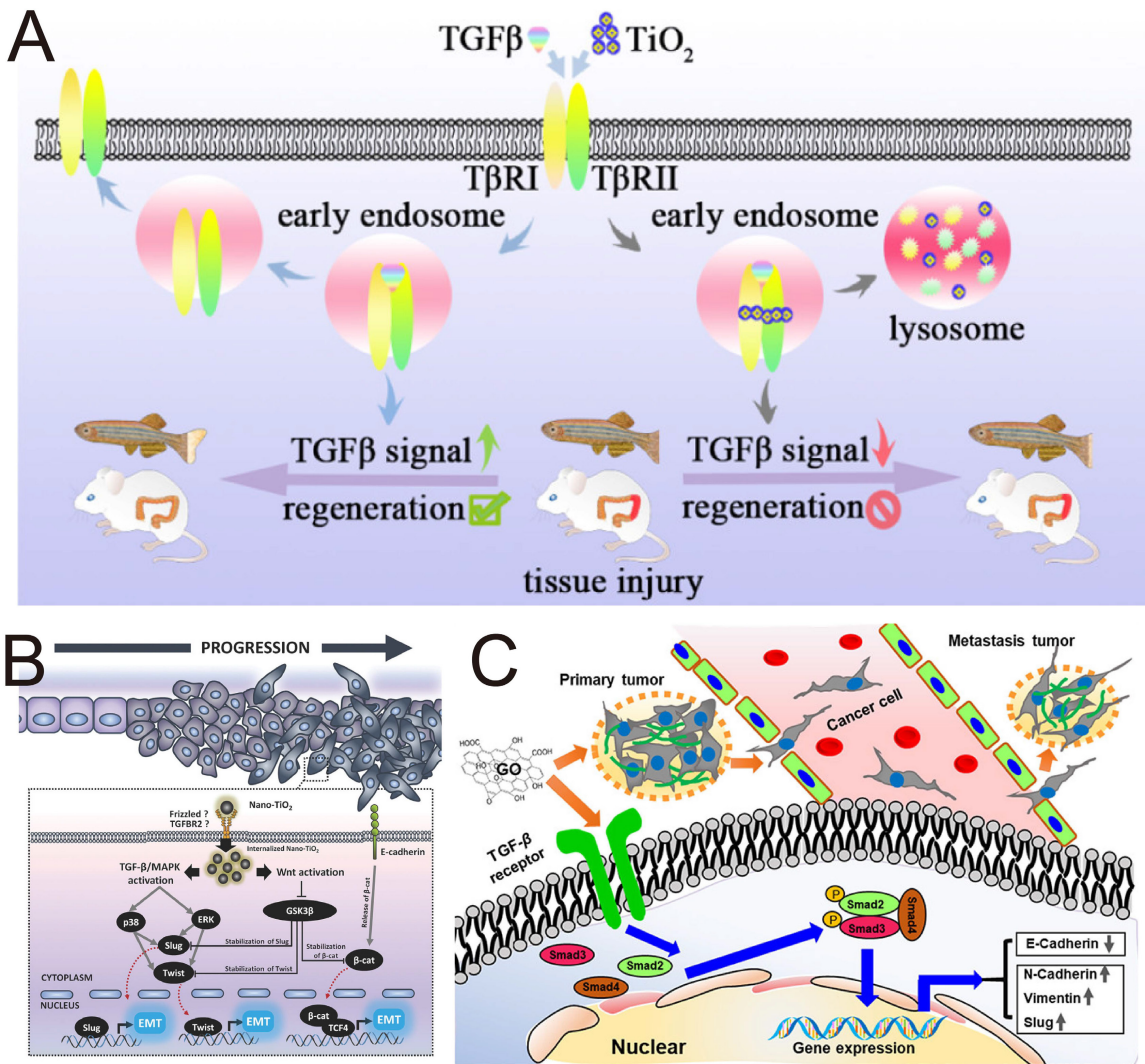
The Janus-faced properties of inorganic NPs in the regulation of TGF-β signaling. (A) Inhibition of EMT by TiO_2_ NPs. Adapted with permission from 140. Copyright 2018, American Chemical Society. (B) TiO_2_ NP-induced cell migration and invasion in intestinal epithelial cancer cells. Adapted with permission from 142. Copyright 2018, John Wiley and Sons. (C) GO nanosheets promote cancer metastasis by promoting TGF-β signaling-dependent EMT. Adapted with permission from 146. Copyright 2020, American Chemical Society.

Titanium dioxide NPs (TiO_2_ NPs) are among the most extensively produced nanomaterials and are widely applied in food production, cosmetics, and pharmaceuticals. Studies have shown that TiO_2_ NPs are capable of inhibit EMT with negligible cytotoxicity toward human lung epithelial A549 cells (**Figure 6A**). In terms of mechanism, TiO_2_ NPs bind to the TGF-β receptors TβRI/II, promote the trapping of TGF-β receptors in the lysosome, and interfere with the phosphorylation of SMAD2/3 140. Zinc oxide (ZnO) NPs are unique metal oxide nanomaterials that have garnered significant attention in the biomedical field due to their ability to produce zinc ions (Zn^2+^) and ROS. Metal ions and ROS act as critical modulators of stem cell physiology and significantly influence their metabolic processes and immunological functions. Recent research indicates that ZnO NPs can efficiently inhibit the production of TGF-β and α-SMA in adipose-derived stem cells, exhibiting significant potential for treating inflammation-related liver fibrosis 141.

##### 4.2.2.2 Inorganic NPs activate the TGF-β signaling pathway

While NPs targeting TGF-β have outstanding tumor-suppressive effects, inorganic NPs can induce prometastasis and side effects by activating TGF-β signaling. Magdiel *et al.* demonstrated that nano-TiO_2_, which is universally added to food products, can promote EMT in colorectal carcinoma through both the TGF-β/MAPK pathway and the Wnt pathway (**Figure 6B**) 142. Analogously, both silica NPs (nano-SiO₂) and hydroxyapatite NPs (nano-HA), which are commonly utilized as food additives, accumulate in colorectal cancer cells and induce an elongated fibroblast-like morphology. This morphological change is also capable of promoting EMT progression. This study revealed that EMT induction is not merely confined to nano-TiO₂ exposure but may represent a broader nanomaterial-induced phenomenon, indicating the possible toxicity and side effects of absorbable airborne nanomaterials in the induction of EMT progression. Similarly, nickel oxide NPs (Nano NiO) have been shown to induce EMT progression through TGF-β1/SMAD pathway in a hepatoblastoma-derived cell line (HepG2), resulting in hepatic fibrosis 143. A similar tumor-promoting phenomenon has also been verified in another study investigating TiO₂ nanofibers. TiO_2_ nanofibers cause lung epithelial cells to obtain an aggressive tumor phenotype with upregulation of HIF-1α, TGF-β and N-cadherin, which serve as angiogenic, profibrotic and EMT markers, respectively 144.

Fe_3_O_4_ magnetic NPs (MNPs) arguably rank among the most extensively researched nanomaterials, particularly in cancer theranostics. Using PEI to guarantee MNPs colloidally stable in cell culture media, Man *et al.* constructed PEI-coated MNPs (PEI-MNPs), which served as a model to determine how the MNPs elicited cellular responses. PEI-MNPs led to tumor progression through the induction of autophagy and the activation of the TGF-β/ NF-κB signaling pathways via the generation of ROS 145.

Depending on many properties, including size, morphology, surface charge, chemical composition and other reactive properties, inorganic NPs might exhibit prometastatic effects and toxicity through TGF-β signaling. Zhu *et al.* reported that low-dose GO nanosheets with a large size range of 400-900 nm (GO-L) and a small size range of 200-600 nm (GO-S) similarly induced remarkable morphological and structural variations in the tumor cell membrane (**Figure 6C**), promoting tumor cell migration and invasion by activating TGF-β signaling and increasing the number of TGF-β receptors; although GO nanosheets induced cytotoxicity at relatively high doses 146. GO activated TGF-β signaling to exert prometastatic effects in a dose-dependent manner. Another study revealed that small-sized (22 nm, 42 nm, and 71 nm) silver NPs (AgNPs) increase the expression of TGF-β, proinflammatory cytokines, and Th1-type and Th2-type cytokines in serum in a concentration-dependent manner and are more active at triggering toxicological or biological responses than large-sized AgNPs (323 nm) 147. The results obtained by Park *et al.* proved that orally administered small-sized AgNPs were more capable of inducing organ toxicity and inflammatory responses than large-sized AgNPs were. Aluminum oxide (AlO) NPs are considered potential immunoadjuvants and drug delivery carrier materials. Manshian *et al.* demonstrated that wire-shaped AlO with a high aspect ratio, but not spherical-shaped AlO NPs, leads to tumor-associated inflammation and metastasis in animal models through the activation of the NLRP3 inflammasome and high expression of TGF-β 148. All these findings indicate that the side effects of nanomaterials should be considered in the development of nanomedicines. The physicochemical properties of inorganic NPs (e.g., concentration, size, and morphology) are key factors influencing their effects on TGF-β. Future research should focus specifically on how changes in these physicochemical properties affect the bioactivity and safety of NPs.

#### 4.2.3 Hybrid NPs

Owing to the combination of inorganic nanomaterials with organic nanomaterials, hybrid NPs with increased biocompatibility and suspension characteristics have garnered increasing attention in the development of versatile nanosystems for the delivery of TGF-β pathway antagonists (**Table 4**). Owing to their therapeutic and imaging abilities, controlled release, and versatility, hybrid nanosystems are promising for the theranostics of tumors.

Hybrid NPs composed of magnetic components, such as superparamagnetic iron nanocubes (SPIONs) and Fe_3_O_4_ NPs, hold significant potential for magnetic resonance imaging (MRI), hyperthermia of tumors, as ferroptosis inducers, and magnetically driven targeted delivery. Mardhian *et al.* designed and synthesized human relaxin-2 (RLX)-conjugated SPIONs using biochemical conjugation techniques to suppress the differentiation of PSCs into CAF-like myofibroblasts, which improved the chemotherapeutic efficacy of gemcitabine and decreased collagen deposition in PDAC 149. Compared with free RLX, RLX-SPION resulted in a greater reduction in α-SMA expression and more efficacious inhibition of TGF-β-induced pancreatic stellate cell differentiation, which was attributed to the multivalent effect of RLX on SPIONs. Recently, the induction of ferroptosis by iron oxide particles in tumor cells has emerged as a prospective strategy to elicit immunogenicity, adaptive immune responses, and therapeutic outcomes. Zhou *et al.* developed anti-TGF-β antibodies and arginine-glycine-aspartic dimer (RGD2)-conjugated Fe_3_O_4_/Gd_2_O_3_ hybrid NPs loaded with sorafenib (FeGd-HN@Sorafenib@TGF-β-antibody@RGD2, FG-STR) for MRI-guided TME heating 150. Synergistic combination of TGF-β blockade and ferroptosis represents an effective strategy for increasing the response rate of poorly immunogenic tumors to ICIs by modulating the TME; however, their side effects on normal tissues still restrict their clinical application. This nanosystem achieves highly efficient MRI-guided combination therapy through the combination of ferroptosis and spatiotemporal TGF-β blockade.

**Figure 7:**
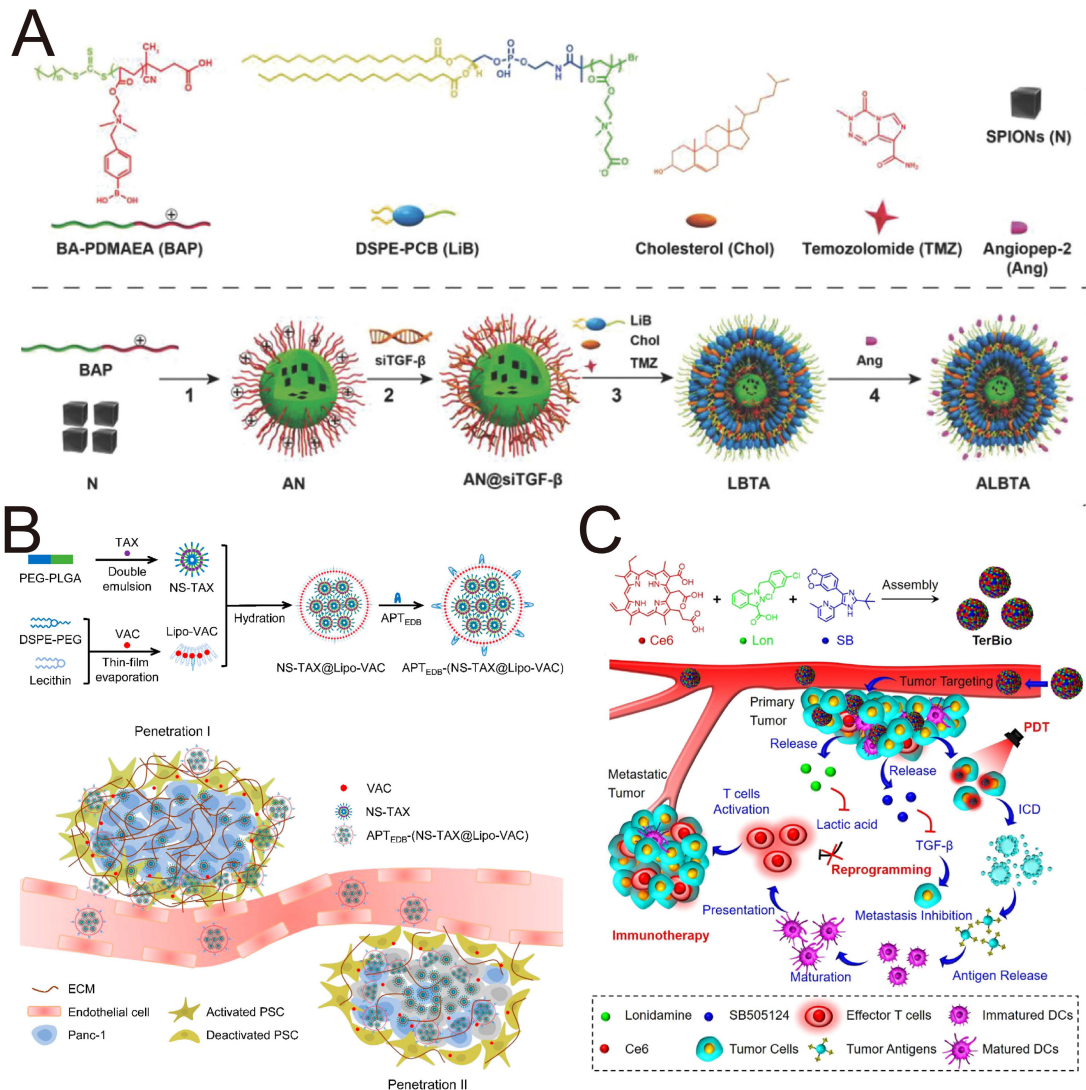
Studies of hybrid NPs, lipid-polymeric NPs and small-molecule NPs targeting TGF-β for cancer treatment. (A) Schematic illustration of the nanotheranostic system (Angiopep LipoPCB (Temozolomide+BAP/siTGF-β), ALBTA) with dual targeting and ROS response to TGF-β RNAi-based immunochemotherapy for glioblastoma. Adapted with permission from 151. Copyright 2018, John Wiley and Sons. (B) Schematic illustration of the size-switchable lipid‒polymer hybrid nanoplatform (APT_EDB_-(NS-TAX@Lipo-VAC) against desmoplastic PDAC. Adapted with permission from 160. Copyright 2021, American Chemical Society. (C) Schematic illustration of the self-assembled carrier-free nanodrug for TGF-β blockade and PDT-amplified immune therapy. Adapted with permission from 163. Copyright 2022, American Chemical Society.

The use of siRNA to regulate the TME for enhanced chemotherapy is promising. Qiao *et al.* developed a hybrid ROS-responsive nanosystem (Angiopep LipoPCB (Temozolomide+BAP/siTGF-β), ALBTA) with the ability to efficiently cross the blood-brain barrier and escape endosomal/lysosomal degradation for TGF-β RNAi-based immunochemotherapy of glioblastoma (**Figure 7A**) 151. Positively charged BAP polymers encapsulating SPIONs were used to complex with siTGF-β and prepare AN@siTGF-βNPs, excluding the “off-target” effect of siTGF-β by the controlled release of siRNA to construct a ROS-labile charge-reversal BAP. Lipid-based envelopes were subsequently coated on AN@siTGF-βNPs, and temozolomide (TMZ) was added to construct LBTA NPs; zwitterionic liposomes protected the siRNA from endosomes/lysosomes. Finally, the peptide angiopep-2 targeting the BBB and glioblastoma cells was conjugated to the NPs to obtain the optimized nanosystem (ALBTA) for accurate MRI and intracranial glioblastoma treatment. This nanosystem crosses the blood-brain barrier via transcytosis and responsively releases drugs and contrast agents based on changes in the local microenvironment, enabling combined immunotherapy and chemotherapy for intracranial glioblastoma.

Au NPs possess extensive biomedical applicability because of their distinct physicochemical properties, such as a tuneable morphology, expansive surface area, amphiphilic nature, and carrier capacity for drug and gene delivery. Wu *et al.* synthesized positively charged cysteamine-functionalized AuNPs to condense a negatively charged siTGF-β1 and then coated S-PEG on the surface to cover the free cysteamine. These AuNP-siRNA nanocomposites delivered siTGF-β1 into implanted tumor cells and protected them from physiological conditions to knockdown TGF-β1 signaling for oncotherapy 152. In addition to delivering nucleic acid drugs, ligand-modified amphiphilic gold NPs can also deliver hydrophobic compounds, such as TGF-β1 inhibitors. Another study by Yang *et al.* revealed that 11-mercaptoundecane sulfonate ligand-modified Au NPs (Amph-NPs) were competent at secluding small-molecule drugs, such as SB525334, within the hydrophobic pockets of their ligand shells 153. This flexible organic ligand shell terminated by hydrophobic methyl and water-solubilizing sulfonate groups not only allows NPs to embed within lipid bilayers to enter cells without toxicity but also provides a methyl group to serve as a reactive site for the subsequent chemical conjugation of antibody proteins. After being further conjugated with anti-CD8 VHH nanobody, targeting antibodies conjugated with Amph-NPs (VHH-NPs) promoted the delivery of SB525334 to T cells accompanied by increased production of both IFN-γ and TNF-α, while free SB525334 did not influence T cell functionality 153. These studies might focus on the intrinsic effects of Au NPs on the TGF-β signaling pathway for the further development of Au-based hybrid NPs.

Cu-based nanomaterials, such as copper oxide, Cu metal-organic frameworks, CuS, and Cu_2_(OH)PO_4_, exhibit enzyme-mimicking properties, photothermal performance, and cancer imaging ability. Cai *et al.* synthesized CuS NPs modified with PEG-anti-TGF-β on the surface to deliver an ataxia telangiectasia mutated (ATM) inhibitor for chemotherapy and CuS-mediated photothermal therapy (PTT). Compared with the cellular uptake in LO2 cells and HepG2 cells, the ability of CuS-ATMi@TGF-β NPs to target hepatocellular carcinoma cells was proven by increased red fluorescence 154. This enhanced cellular uptake and targeting specificity is mediated by anti-TGF-β. In a study of glioblastoma multiforme, Chen *et al.* developed a “one-for-all” hybrid theranostic agent, Cu_2_(OH)PO_4_@PAA, based on polyacrylic acid (PAA)-coated Cu_2_(OH)PO_4_ NPs for MRI-guided PTT, which suppressed the MSH6-CXCR4-TGF-β1 pathway and its downstream factors in glioblastoma multiforme 155. Most investigations have concentrated on increasing the expression of MSH6 to increase the efficacy of temozolomide. Nevertheless, the function and signaling pathway of MSH6 have not yet been considered. This study demonstrated that the oncogenic MSH6-CXCR4-TGF-β1 feedback loop represents a promising therapeutic target in GBM and that the PTT mediated by Cu₂(OH)PO₄ is related to the MSH6-CXCR4-TGF-β1 axis.

Most patients with PDAC are not eligible for surgical resection, and the dense tumor stroma contributes to a poor response to current chemotherapy and radiotherapy. IRE has emerged as a novel ablation modality used in the clinic for PDAC patients, but IRE remains associated with a persistent risk of tumor recurrence. The porous structure and easily modified surface of mesoporous silica NPs (MSNs) provide a robust platform for loading diverse therapeutic agents. Peng *et al.* fabricated glutathione-responsive degradable mesoporous silica NPs to deliver SB525334 for reprogramming tumor-associated neutrophils against PDAC 156. The regulation of the immune TME enhanced the therapeutic response of tumors to irreversible electroporation and anti-PD1 therapy. This study addresses the issue of tumor recurrence following irreversible electroporation by blocking TGF-β and PD-1 expression, providing valuable clinical insights for the treatment of PDAC.

MSNs also provide a nanoplatform for multimetal nanozymes with high peroxidase-like and catalase-like activity. Xu *et al.* constructed SB-505124-loaded PEGylated iron manganese silicate NPs (IMSN-PEG-TI) incorporating Mn^2+^ and Fe^2+^ for nanozyme-mediated catalytic therapy 157. The blockade of TGF-β modulated immune TEM, inducing macrophage polarization towards M1 phenotype. This nanozyme catalyzes the production of H_2_O_2_ for ferroptosis mediated tumor therapy. The development of nanozymes to modulate the TME holds significant research value and clinical relevance for improving the efficacy of nanozyme-based catalytic tumor therapy.

#### 4.2.4 Lipid-polymeric NPs

Lipid-polymeric NPs represent a class of nanocarriers characterized by an internal polymeric core enveloped by a multilayered outer lipid shell. This unique architecture endows them with the versatile ability to encapsulate diverse payloads, encompassing both hydrophilic and hydrophobic therapeutic agents. For instance, a hybrid core-shell nanoplatform was developed to codeliver both the hydrophobic inhibitor SB-505124 and the hydrophilic protein cytokine IL-2 for enhanced tumor immunotherapy 158. The inhibitor-loaded methacrylate-modified β-cyclodextrins and IL-2 were encapsulated in an interlaced polymer matrix with a PEGylated liposomal coating, which enabled the sustained release of TGF-βi and IL-2 for seven days 158. Blocking TGF-β signaling and stimulating the activation of T cell responses via IL-2 had a synergetic anticancer effect on a B16/B6 melanoma mouse model through the activation of both innate and adaptive immune responses.

Lipid-polymeric NPs have risen as powerful platforms for mRNA delivery; however, the endolysosomal/lysosomal barrier and dense ECM hinder the penetration and delivery efficiency of lipid-polymeric NPs. Young *et al.* constructed novel lipid formulations (LNP) that leverage esomeprazole (ESO) to promote the escape of mRNA from endo-/lysosomes 159. As a proton pump inhibitor, ESO hinders the acidification of the endo-/lysosomal pH and destroys membrane integrity, which induces endo-/lysosomal escape. Furthermore, the ESO-loading LNP remodel the ECM through the TGF-β/SMAD signaling pathway, contributing to the deep penetration of both LNP and mRNA. The use of ESO-loading LNP formulations for mRNA delivery allows for both increased local expression of the mRNA and effective immune activation.

The sequential release of TGF-βi and drug is important for the cascade penetration strategy to achieve the anticipated effect. While small NPs exhibit superior penetration into deep tumor regions, their propensity for rapid macrophage-mediated clearance presents a significant therapeutic limitation. An optimal strategy was developed to address this dichotomy using a size-switchable lipid-polymer hybrid nanosystem (APT_EDB_-(NS-TAX@Lipo-VAC) to achieve drug penetration and the sequential release of vactosertib (VAC) and paclitaxel (TAX) in the desmoplastic stroma (**Figure 7B**) 160. A hydrophobic layer of smaller PEG-PLGA nanospheres (NS-TAX) was loaded with TAX and subsequently loaded into larger liposomes, which delivered VAC within a phospholipid bilayer (Lipo-VAC), thereby constructing the nanosystem NS-TAX@Lipo-VAC. The liposomes were functionalized with APT_EDB_ peptide targeting fibronectin extra domain B (EDB), which is highly expressed in the ECM, to achieve targeting and long retention in the tumor. Larger NPs functionalized with APT_EDB_ peptide are targeted precisely to the tumor ECM, and the earlier release of VAC, which is accompanied by the collapse of liposomes, not only decreases the size of the nanosystem but also inactivates PSCs and remodels the ECM via TGF-β blockade to promote the subsequent penetration of TAX, thus astricting pancreatic tumor xenografts more efficiently than free drugs do. This cascade permeation strategy, which employs a stroma-regulable and size-switchable nanosystem, holds significant potential for the treatment of stroma-rich tumors.

#### 4.2.5 Small-molecule NPs

Self-assembled nanodrugs using small-molecule drugs represent a promising alternative that can integrate multiple therapeutic agents within a single platform without additional toxicity or adverse effects associated with conventional carriers. Oleanolic acid (OA), a naturally pentacyclic triterpene found in plants, possesses self-assembly and gelling properties and antitumor effects for the development of carrier-free nanodrugs. Moreover, OA suppressed TGF-β1/SMAD signaling by binding to TβRI, inactivating the CAFs and remodeling the ECM. Thus, we fabricated rigid NPs (OA-NP) and flexible nanogels (OA-NG) to elucidate the effects of elasticity on antitumor effects and ECM reconstruction 161. Among the different OA formulations, OA-NG strongly inhibited the expression of α-SMA, contributing to prolonged blood circulation and deep penetration of flexible OA-NG. Our group further developed a small-molecule nanodrug based on OA nanogels incorporating the sonosensitizer rose bengal (RB) and the photothermal agent methylene blue (MB) for the inactivation of CAFs via TGF-β blockade and PTT/SDT-assisted immunotherapy 162. Self-assembled ORM nanogels exhibit three-pronged deep penetration into the TME, in which they cooperate synergistically with SDT or PTT to suppress TGF-β1/SMAD signaling and inactivate CAFs.

The excessive accumulation of TGF-β and lactic acid (LA) is strongly correlated with the immunosuppressive TME. Zhao *et al.* constructed self-assembled carrier-free bioregulators (TerBio) that incorporate the photosensitizer Ce6, SB505124 and the LA inhibitor lonidamine for immunosuppressive TME remodeling by blocking the activity of TGF-β and LA (**Figure 7C**) 163. With a high drug entrapment efficiency, carrier-free TerBio exhibits increased tumor accumulation and deep penetration. The combination of the ICD effect induced by PDT and the immunoactivating TME synergistically amplifies the efficacy of immunotherapy against colorectal cancer. Compared with the use of inert carrier materials, the use of carrier-free nanodrugs with simple compositions through facile preparation can overcome biosafety challenges, such as difficulties in carrier degradation and metabolic clearance.

#### 4.2.6 Cell membrane-coated biomimetic NPs

Cell membrane-coated NPs have risen as a burgeoning nanocarriers (**Table 5**) because of their improved biocompatibility, lower immunogenicity and precise tumor-targeting capabilities. Characterized by a synthetic or self-assembled nanoparticulate core encapsulated within a naturally derived cell membrane coating, cell membrane-coated NPs can elicit diverse desirable biological effects due to the inherent, multifunctional properties inherited from their source cells.

Immune cell membrane-coated NPs are constructed with the ability to identify antigens for precise targeting, and these NPs have various functions, which are derived mainly from the intrinsic function and surface proteins of immune cells. Building on this principle, Kim *et al.* developed an innovative therapeutic strategy involving macrophage membrane-coated NPs (Mφ-SDN) loaded with SD-208 174. These NPs selectively targeted TAMs, metastasis-associated macrophages (MAMs) and cancer cells, effectively suppressing tumor metastasis and reprogramming the immunosuppressive TME by blocking M2-type macrophage differentiation. Wang *et al.* fused the macrophage membrane and conjugates of anti-PDL1 and PEGylated phospholipids to coat gold nanocages and loaded LY2157299 into the core, which was developed to form a nanocomposite (GNC-Gal@CMaP) 175. In response to contributions from the cell membrane and anti-PDL1 modification, GNC-Gal@CMaP exhibited promising tumor-specific targeting ability and could avoid elimination by the reticuloendothelial system. The suppression of TGF-β not only reversed the immunosuppressive TME but also suppressed TGFβ-mediated thermoresistance by downregulating the expression of heat shock proteins 70 and 90 in connection with the thermoresistance of tumor cells. These nanocomposites facilitate low-temperature PTT-induced ICD, promoting the activation of antigen-presenting cells and the subsequent recognition of tumor-specific effector T cells, thereby potently suppressing both primary and metastatic tumor progression through the dual suppression of PD-1/PD-L1 and TGF-β.

**Figure 8:**
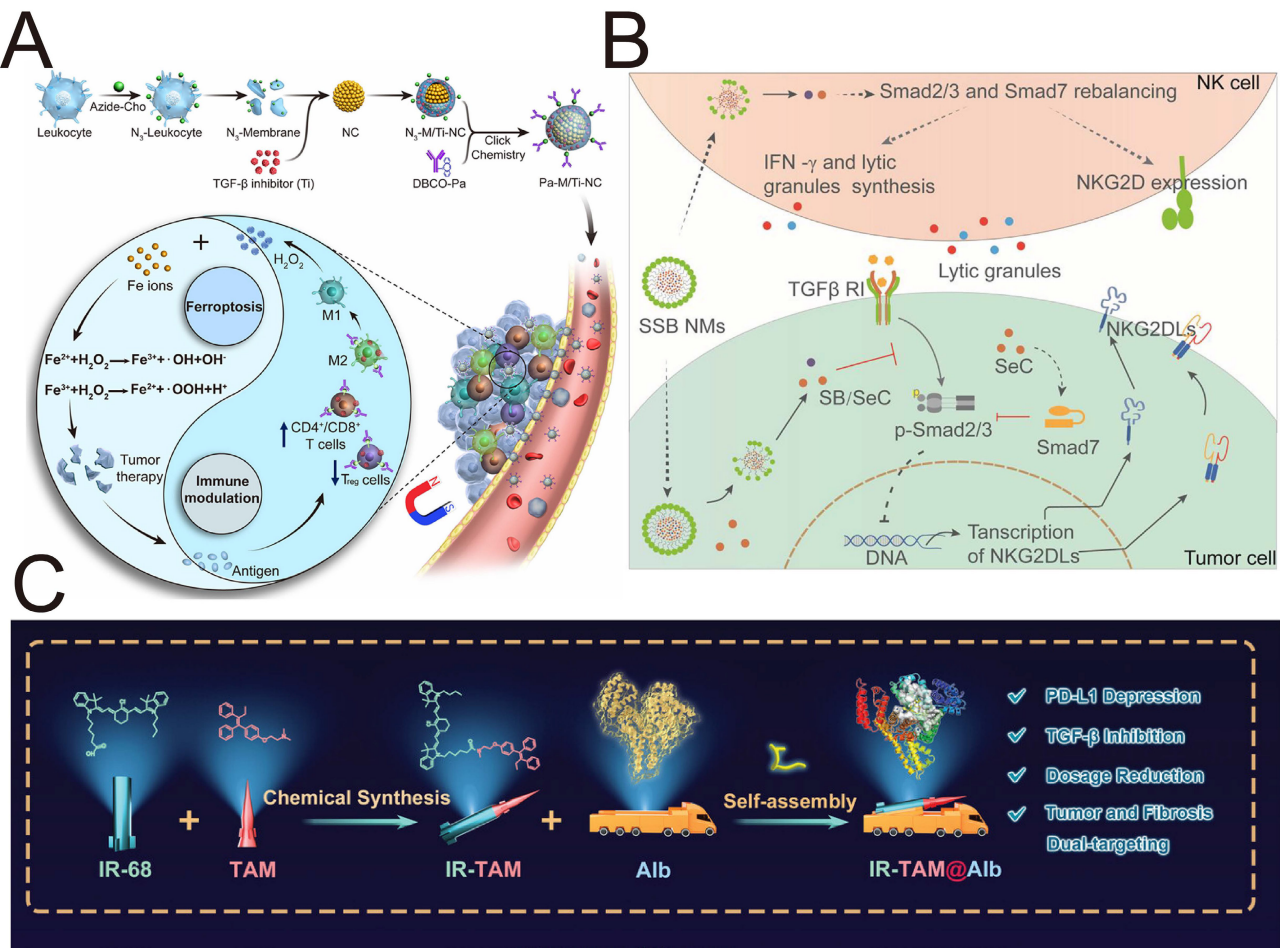
Studies of biomimetic NPs, immune cell therapy and protein-based NPs targeting TGF-β for cancer treatment. (A) Schematic illustration of the biomimetic magnetosome for ferroptosis/immunomodulation-mediated tumor treatment. Adapted with permission from 176. Copyright 2019, American Chemical Society. (B) Schematic illustration of proposed signaling pathway triggered by SSB NMs for NK cell-based cancer immunotherapy. Adapted with permission from 182. Copyright 2020, American Chemical Society. (C) Schematic illustration of the IR-TAM@Alb nanosystem for enhancing radioimmunotherapy to prevent fibrosis development induced by dual TGF-β/PD-L1 blockade. Adapted with permission from 187. Copyright 2024, John Wiley and Sons.

Biomimetic Fe_3_O_4_ magnetic nanoclusters coated with anti-PD-1 antibody-conjugated leukocyte membranes were constructed for tumor-targeted delivery of the SB-505124 hydrochloride with long circulation (**Figure 8A**) 176. Ferroptosis induced by Fe ions and intratumoral H_2_O_2_ boosts the immunogenicity, and the combination of ICIs and TGF-βi results in a cyclical immune pattern 176. Using different tumor models of breast cancer and melanoma, this combination strategy of immunomodulation and ferroptosis was shown to have superior therapeutic performance without tumor recurrence or metastasis.

The application of tumor antigen-primed DC membranes to coat NPs ensures that targeted delivery contributes to the individual capacity of DCs to trigger antitumoral T cell responses. Kim *et al.* designed lipid biomimetic NPs (T-DCNP) by integrating autologous neoantigen-primed DC membranes with liposomes loaded with SB525334 within their lipid bilayers using a coextrusion strategy 177. The encapsulation of TGF-βi in the lipid bilayer protected the inhibitor from degradation, with an outstanding ability to reverse the immunosuppressive TME. The tumor-derived antigen-primed DC cell membrane endows T-DCNP with patient-personalized antitumor immune responses for postsurgical immunotherapy, enabling targeted attachment to T cells by effective fusion and native targeting. T-DCNP reshaped the TME with increased infiltration of CD8^+^ T cells and dual suppression of PD-1 and TGF-β, resulting in significant suppression of the growth of primary and distant tumors and protection against tumor rechallenge.

In contrast to the strategies for delivering TGF-βi in the aforementioned studies, a subsequent study by An *et al.* utilized T cell membranes with high expression of TβR and PD-1 receptors to exhaust TGF-β and block PD-L1 within tumor cells 178. After being coated with the EL4 T cell membrane, a nanogenerator (CaNP@ECM) based on CaO_2_ NPs efficiently accumulated at the tumor site through the binding of the adhesion protein LFA-1 expressed on the EL4 cell membrane to inflamed endothelial cells in the TME. This T cell-mimicking Ca^2+^ nanogenerator provides diverse benefits for cytotoxic lymphocyte cell-mediated chemoimmunotherapy through the simultaneous induction of ICD effects, inhibition of tumor cell glycolysis and reversal of the immunosuppressive TME.

Recent advancements have demonstrated that the tumor cell homing mechanism offers a targeted delivery approach for NPs functionalized with tumor cell membranes. Song *et al.* developed a tumor cell membrane-coated trimetallic nanozyme (LY-Au@Pt@Rh-CM) composed of three metals (Au, Pt, and Rh) to deliver LY2157299 within the large pore space for photothermal/immunomodulation-boosted catalytic therapy. Owing to the homotypic targeting property of the cancer cell membrane, the nanocomposites achieved the targeted delivery of LY2157299 to tumors without premature leakage. The combination of the polarization of macrophages towards the M1 phenotype by TGF-βi and nanocomposites with dual peroxidase- and catalase-like activities achieved superior therapeutic effects upon laser irradiation 179.

For efficient treatment of colorectal cancer, efficient delivery of therapeutic agents to the intestinal tract is important. Decorating NPs with bacterial membranes helps them cross the intestinal mucus barrier with deep penetration, prolonged retention and controlled release. Li *et al.* fabricated a *S. aureus* membrane-coated nanosystem (LNSM-RS) with a core of X-ray-excited lanthanide-doped scintillators and a hollow macroporous silica shell to entrap SB525334 and NO precursors—Roussin's black salt 180. By precisely targeting expressed Protein A in the TME of colorectal cancer through the *S. aureus* membrane coating method, LNSM-RS exhibited targeted accumulation and retention within the tumor site in an orthotopic colorectal tumor model, whereas the fluorescence signal of LNS-RS without the *S. aureus* membrane coating could not be detected during *in vivo* imaging. The generation of hypertoxic peroxynitrite (ONOO^-^) by lanthanide-doped scintillators upon X-ray irradiation works synergistically with GSH-responsive released SB525334, inducing tumor-associated neutrophil polarization towards the antitumor N1 phenotype for radiotherapy-based synergistic therapy.

Cell membrane-coated biomimetic NPs with superior biocompatibility and prolonged circulation represent a promising strategy for specifically delivering TGF-βi to tumors. The lipid bilayer also allows for the flexible codelivery of other therapeutic agents. However, this approach faces considerable challenges related to the complexity of manufacturing, including complex isolation and fusion processes, poorly reproducible production, and variability in encapsulation efficiency. Despite these drawbacks, the ability to precisely deliver these critical signaling inhibitors while minimizing systemic toxicity makes cell membrane-coated biomimetic NPs highly attractive platforms in advanced nanomedicine.

#### 4.2.7 Immune cell therapy

Immune cell therapy has risen as a revolutionary strategy against tumors, utilizing the host immune system to defeat cancer cells. In contrast to conventional chemotherapy and radiotherapy, immunotherapy performed superior antitumor treatment with reduced side effects and long-term protection from recurrence. Among the most promising strategies are CAR-T cell therapy, NK cell therapy, and tumor-infiltrating lymphocyte (TIL) therapy. These approaches have shown remarkable efficacy in hematologic malignancies and are being actively explored for solid tumors.

CAR-T cell therapies have garnered enormous attention for treatment of solid tumors; however, the immunosuppressive TME derived from TGF-β and other modulators has limited the therapeutic effects of CAR-T cell therapies. PTT has risen as a minimally invasive strategy for tumor treatment, utilizing the photothermal conversion capabilities of NPs to generate localized hyperthermia upon laser irradiation, improve the tumor stromal barrier, and activate tumor immunity. Tang *et al.* developed an amphipathic nanodelivery system (HES-PCL NPs) to codeliver the LY2157299 (LY) and the photothermal agent indocyanine green (ICG) for the treatment of lymphoma 181. The photothermal response of LY/ICG@HESPCL NPs accompanied by the degradation of ICG is beneficial for the responsive release of LY *in vivo*. TGF-β blockade combined with PTT reverses the TGF-β-mediated suppression of CAR-T cell trafficking by upregulating the expression of CXCL9/10/11 and its cognate receptor CXCR3 while simultaneously mitigating T cell exhaustion and promoting effector memory T cell differentiation 181. Compared with single CAR-T cells, synergistic therapy with these nanosystems and CAR-T cells resulted in 2.4-fold greater antitumor effects and 2.7-fold greater relapse suppression rates during the treatment period. This study revealed that the combination of TGF-β blockade with PTT enhances the long-term antitumor effect of CAR-T cells.

NK cell adoptive therapy stands as a prospective strategy to circumvent the limitations of traditional therapies; nevertheless, the antitumor effect of NK cells is significantly impacted by the immunosuppressive TGF-β. Liu *et al.* developed a nanoemulsion system (SSB NMs) via convenient emulsification to encapsulate SB505124 (SB) and selenocysteine (SeC), combining TGF-β blockade and adoptive NK cell-based immunotherapy for TNBC treatment (**Figure 8B**) 182. Benefiting from the nanoemulsion, the poor stability and inferior solubility of SB and SeC were attenuated, with good stability for 4 weeks. When SSB NMs were combined with adoptive NK92 cell-based immunotherapy, SSB NMs increased the killing potency and intratumor infiltration of NKs, resulting in the upregulation of NKG2DL and the inhibition of TGF-β/SMAD2/3 signaling, highlighting the application and mechanism of action of NK cells in adaptive therapy for TNBC treatment.

While the above study combines NPs and NK cells to achieve TGF-β blockade and adoptive cell therapy, subsequent work utilizes polymeric NPs encapsulating TGF-βi to chemically reprogram NK cells for adoptive cell therapy. Choi *et al.* 183 developed polymeric nanogels encapsulating galunisertib to chemically prime NK cells, termed Nano-Chem_NK, leading to the reversion of the immunosuppressive TME for efficient treatment of TGF-β-secreting cancers. This strategy utilized hydrophilic negatively charged HA to encapsulate hydrophobic galunisertib through self-assembly, whose surface was coated with positively charged 25K PEI. These nanogels reverse the suppressive TME by chemically priming NK cells and blocking TGF-β activity.

Screening competent targeting agents for the precise delivery of TGF-βi to transferred T cells may reveal differences in their internalization and therapeutic efficacy. Zheng *et al.* developed two liposomes, anti-Thy1.1-Lip and anti-CD45-Lip, with two different receptors to deliver the TβR-Ⅰ inhibitor SB525334 to T cells for adoptive cell therapy 184. Compared with internalized receptor (CD90 or Thy1)-targeted liposomes, T cells preloaded with liposomes *in vitro* targeting noninternalizing receptor (CD45) induced higher granzyme expression in T cells and greater donor T cell infiltration of B16F10 melanoma tumors 184. In contrast, when these two different ligands, Thy1 and CD45, were used to direct liposomal TGF-β to lymphocytes, anti-Thy1.1 liposomes enhanced the antitumor effect 184. These findings potentially underscore the premier donor cell specificity exhibited by Thy1 than by CD45, highlighting the critical necessity of targeting TGF-β specifically to effector T cells rather than focusing on other immune cell populations.

The combination of immune cell therapy with a TGF-β blockade strategy represents an emerging and promising direction for cancer treatment. TGF-β signaling within the TME promotes immunosuppression, contributing to therapeutic resistance and tumor progression. By codelivering TGF-βi alongside adoptive cell therapies, this synergistic approach can potentially reverse immunosuppression, improve T cell activity, and increase tumor infiltration. DDSs offer a powerful platform for this combinatorial strategy, enabling the targeted and controlled release of both therapeutic cells and inhibitors. This integrated nanotechnology-based approach holds significant potential to advance the clinical translation of potent and durable cancer immunotherapies.

#### 4.2.8 Protein-based delivery systems

Protein-based drug carriers, such as albumin, ferritin, legumin and gelatin, have risen as prospective platforms for drug delivery. Protein-based platforms can protect loaded drugs from enzymatic degradation and renal clearance. Albumin, a highly soluble protein, possesses a three-dimensional architecture comprising both hydrophilic and hydrophobic domains. Luo *et al.* utilized dithiothreitol (DTT) to cut the disulfide bonds of human serum albumin (HSA), and the obtained unfolded HSA was self-assembled with hydrophobic perfluorotributylamine (PFTBA) to develop NPs (PFTBA@HSA) 185. This unfolded HSA developed using the “unfolding and self-assembly” strategy has a preeminent platelet affinity and delivery capacity, but native HAS does not. Moreover, a dose-dependent elevation in platelet affinity was detected as the degree of unfolding of HSA progressively increased. Compared with other cells, platelets are crucial but devalued carriers of 40-100 times more TGF-β and serve as the dominant source of circulating TGF-β upon exposure to the TME 186. Engineered HSA-coated PFTBA NPs were meticulously designed to exhibit optimal platelet-targeting capabilities, with robust inhibitory effects on platelet TGF-β-mediated tumor metastasis and NK cell immune surveillance.

Radiotherapy has a limited ability to modulate exogenous hypoxia and the immunosuppressive TME; moreover, the increased expression of PD-L1 and TGF-β after radiotherapy further weakens its antitumor efficacy. Treatment with tamoxifen, an antioestrogen drug designed for estrogen receptor-positive breast cancer, can interfere with mitochondrial metabolism regardless of estrogen receptor status and induce the downregulation of both TGF-β and PD-L1. Thus, Zhou *et al.* conducted a mitochondrial-targeted nanosystem (IR-TAM@Alb) by utilizing albumin to deliver tamoxifen-IR68 (heptamethine cyanine dyes) conjugate for efficient radiotherapy to prevent fibrosis development (**Figure 8C**) 187. This mitochondrion-targeted heptamethine cyanine increase the efficacy of radiotherapy via synergistic integration of dual TGF-β/PD-L1 blockade. Owing to its ability to encapsulate albumin and target mitochondria, this targeted nanosystem significantly suppressed radiation-induced fibroblast activation by blocking TGF-β, whereas the nanosystem without mitochondria-targeting dyes failed to attenuate radiation-induced fibrosis at the same dose.

Protein-based carriers possess superior biocompatibility and low immunogenicity, making them highly appropriate for delivering TGF-β pathway antagonists. Their inherent biodegradability and natural origin minimize adverse immune reactions, which is a significant advantage for sustained therapeutic applications. Furthermore, the functional groups on proteins allow for precise chemical modification, enabling the targeted, controlled release of TGF-β pathway antagonists in specific tissues to reduce systemic side effects. However, structural instability under certain physiological conditions and the risk of enzymatic degradation before the target site is reached remain unknown. Despite these limitations, the versatility and safety profile of protein-based carriers position them as promising vehicles for the optimized delivery of TGF-β therapeutic agent.

## 5. Challenges and opportunities

Currently, research on TGF-β-targeting NPs predominantly focuses on the tumor-promoting role of TGF-β, the design of multifunctional NPs to block TGF-β for tumor treatment or the examination of how inorganic NPs may exert protumor effects through the TGF-β signaling pathway. Nevertheless, studies addressing the tumor-suppressive function of TGF-β, particularly in the context of NP-based interventions, are scarce. This one-sided approach limits the potential for precise modulation of TGF-β, which remains an underexplored challenge in nanomedicine because of its dual role in different stages of tumors.

Several inorganic NPs, such as nano-TiO_2_, nano-SiO₂, nano-HA, nano NiO, Fe_3_O_4_ magnetic NPs, GO nanosheets, AgNPs, and AlO NPs, have been shown to induce prometastatic effects and side effects through the TGF-β signaling pathway. These NPs induce an aggressive tumor phenotype through EMT and an elongated fibroblast-like morphology with high expression of EMT and profibrotic markers, such as HIF-1α, TGF-β, α-SMA, E-cadherin and N-cadherin. A proactive design approach focusing on the intrinsic properties of the material and surface engineering is essential for mitigating the risk of TGF-β/EMT activation. The hydrophilic PEG layer minimizes protein absorption, preventing the formation of a protein corona that can trigger downstream TGF-β signaling. Coating INPs with cell membranes from red blood cells or platelets can significantly reduce immune recognition and subsequent profibrotic signaling cascades. Standardized *in vivo* detection methods for early prometastatic signals are crucial for evaluating the safety of inorganic and hybrid NPs. Immunohistochemical staining of primary tumor sections for EMT and pro-fibrotic markers should be executed to distinguish the aggressive phenotype of tumors. Periodically collected blood samples can be analyzed for ctDNA carrying tumor-specific mutations or for the presence of circulating tumor cells that express mesenchymal markers. An increase in the levels of these circulating biomarkers is a strong early indicator of increased metastatic activity.

To date, only one phase I clinical study has investigated the efficacy and biosafety of a polypeptide NP-encapsulated siRNA, STP705, targeting TGF-β1 and COX-2 by subcutaneous administration for localized fat reduction 190, but no clinical trials have been conducted on NPs targeting TGF-β for malignant tumors. Precise modulation of TGF-β's dual functions during tumor progression will boost clinical research and the translation of TGF-β-targeted NPs.

Notably, TGF-β signaling is not uniformly active throughout tumor tissue but displays significant regional variation. Hypoxic cores often exhibit upregulated expression of TGF-β, which induces EMT and stemness. In contrast, invasive tumor cells utilize high TGF-β levels to drive metastasis and immune suppression, whereas perivascular regions may use this pathway for angiogenic signaling. This spatial gradient critically remodels the TME: it recruits and polarizes immunosuppressive cells to specific niches, simultaneously driving the deposition and cross-linking of ECM components by CAFs, leading to heterogeneous stromal stiffness. This physical and biochemical heterogeneity creates a biological barrier, impairing the deep penetration of therapeutics and NPs. Consequently, this landscape highlights a pivotal future direction for the development of intelligent nanodelivery systems that respond to localized TGF-β dynamics caused by hypoxia, enzymes or other stimuli associated with these specific niches.

Additionly, the clinical translation of TGF-β-targeting NPs faces multiple challenges, including unstandardized preclinical assays, manufacturing complexities, regulatory hurdles, and safety concerns. Future research must address three critical challenges and translational bottlenecks to drive the clinical translation of nanomedicine. First, the safety of the material and clear standards for material biodegradation must be prioritized. These standards involve defining acceptable degradation timelines and systematically characterizing and quantifying degradation products, ensuring that they are nontoxic and efficiently cleared. The degradation timelines and systematic characterization and quantification of degradation components should be studied to ensure the biosafety of the material. Currently, only a few studies have systematically investigated the organ-specific toxicity of nanomaterials, and more research should focus on the toxic side effects of carrier materials that promote tumor growth or damage major organs and whether the toxic side effects are caused by the TGF-β-mediated pathway. During the clinical translation of inorganic or hybrid NPs targeting TGF-β, special attention must be given to the potential of these NPs to promote tumor progression via the TGF-β pathway.

Furthermore, preclinical protocols must be rigorously standardized. Longitudinal biodistribution studies should quantify the accumulation of NPs in both tumors and healthy organs over extended periods using established imaging modalities. Comprehensive organ-specific toxicity assessments are crucial for evaluating the expression of immune and fibrotic markers using histopathology. Critically, a dedicated screening platform using diverse *in vivo* models must be implemented to proactively evaluate the risk of TGF-β inhibition potentially enhancing tumor progression in certain contexts.

Finally, robust and validated manufacturing processes are nonnegotiable for investigational new drug (IND) applications. Numerous novel nanoformulations fail to enter clinical trials because complex, laboratory-scale synthesis cannot be scaled up with sufficient reproducibility, purity, or yield for clinical batches. Therefore, the investigation of scalable, reproducible synthesis methods for the development of multifunctional NPs is crucial for IND applications. Rigorous control of critical quality attributes (CQAs), such as the particle size, polydispersity index, drug loading efficiency, and surface functionalization, is pivotal for advancing the application of TGF-β-targeting nanomedicines in the clinic.

## 6. Conclusions

It is indispensable to clarify the dual functions of TGF-β in tumors and to utilize nanotechnology for precise delivery for improving both the treatment effectiveness and biosafety of TGF-β therapeutic agent. The targeted and stimuli-responsive NPs improve the treatment effectiveness of TGF-β pathway antagonists while enabling their precise controlled release, which promotes deep penetration of the NPs through the modulation of the TME. This nanoplatform also serves as a robust tool for the combined and sequential administration of TGF-β pathway antagonists with other therapeutic agent, such as chemotherapeutics, phototheranostic agents, ICIs and immune cell therapies. These advances are expected to facilitate the initiation of clinical trials and provide novel strategies for efficient cancer treatment. Notably, inorganic NPs play dual roles in regulating the TGF signaling pathway, as they may suppress or facilitate tumor progression. Attention must be given to the potential adverse effects and biosafety of nanomaterials to accelerate the clinical translation of NPs and TGF-β pathway antagonists.

## Figures and Tables

**Table 1:** Sequential combination of nanoencapsulated TGF-β pathway antagonists and versatile NPs by a two-wave strategy.

Sequential combination strategy	First-wave NPs loaded with TGF-β pathway antagonists	Second-wave NPs	Cancer cell type	Model	Outcome	Ref.
To increase the efficacy of chemotherapy	PEI/PEG-coated mesoporous silica NP loaded with LY364947	Liposome loaded with gemcitabine	BxPC3	Female BALB/c nude mice	Two-wave approach improves the delivery efficacy of the gemcitabine by overcoming stromal barrier.	106
	CREKA peptide modified PEG-PLA NPs loaded with α-mangostin	CRPPR peptide modified micelle loaded with triptolide	PANC-1	BALB/c nude mice	Sequential delivery strategy can reduce ECM production by inactivating CAFs through TGF-β/SMAD signaling, increasing the efficacy of chemotherapy.	107
	PEGylated liposomes loaded with salvianolic acid B	Docetaxel-loaded PEG-modified liposomes	4T1, NIH3T3	Female BALB/c mice	Nanosystem encapsulating salvianolic acid B remodels the TME and enhances the efficacy of NPs loaded with docetaxel.	112
	Lipid NPs loaded with siTGF-β	Cabazitaxel-loaded albumin NPs	A549	Female BALB/c nude mice	Combination of chemotherapy and TGF-β blockade perform superior effect against paclitaxel-resistant NSCLC.	113
	Albumin-bound triphenylphosphine tamoxifen conjugates	Albumin-bound paclitaxel	4T1	Female BALB/c mice	Nanosystem loaded with tamoxifen induces codepression of PD-L1 and TGF-β, boosting the chemo-immunotherapy of paclitaxel.	103
To enhance the efficacy of molecular targeted therapy	Fraxinellone-loaded CGKRK peptide-modified PEG-PLA NPs	Lipid-coated calcium phosphate NPs for condensing KARS siRNA	PANC-1	BALB/c nude mice	The pro-blockade of TGF-β leads to the inactivation of CAFs, the reduction of M2 macrophage polarization, and normalization of vascular system, opening the new avenue for the subsequent silencing KRAS mutation.	108
To facilitate the efficacy of immunotherapy	PEGylated liposome coated with protamine for codelivery of siTGF-β and HA	Mannose-modified lipid-calcium-phosphate loaded with Trp2 peptide and CpG oligonucleotides	B16/F10	Female C57BL/6 mice	Targeted silencing of TGF-β in TME enhances the efficacy of the vaccine in malignant melanoma.	109
	Fraxinellone	Fraxinellone-loaded AEAA-modified nanoemulsion	BPD6	Female C57BL/6 mice	Fraxinellone nanoemulsion reverses the immunosuppressive TME and facilitates the therapeutic vaccination in desmoplastic melanoma.	110
Combination of TGF-β pathway antagonists and vascular disrupting agent	LY2157299-loaded mPEG_5k_-*b*-PLA_5k_ and maleimide- PEG_5k_-*b*-PLA_5k_	Poly(L-glutamic acid)-graft-poly(ethylene glycol)/combretastatin A4 conjugate	4T1	Female BALB/c mice	The cooperation of TGF-β inhibition and vascular disrupting agents significantly suppress the tumor growth and metastasis.	111

TGF-β: transforming growth factor-β; NPs: nanoparticles; PEI: polyethyleneimine; PGA: polygalacturonic acid; PLA: polylactide; HA: hyaluronic acid; TME: tumor microenvironment; ECM: extracellular matrix; CAFs: cancer-associated fibroblasts; NSCLC: non-small cell lung cancer.

**Table 2:** Summary of polymer-based NPs targeting TGF-β for cancer treatment.

Characteristic	TGF-β pathway antagonists	Nanoparticle/carriers	Tumor cell type	Model	Outcome	Ref.
Enzymatic stimuli	LY2157299	HES-PLA NPs for codelivery of DOX and LY2157299	4T1	Female BALB/c mice	This codelivery nanosystem eliminates the insufficient chemotherapy promoted metastasis.	114
	LY2109761	Nanopolyplex self-assembled by DSPE-PEG-plectin-1 peptide and CPI-613 conjugated PEG for delivery of LY2109761	PANC-1	BALB/c nude mice	These nanopolyplexes achieve stromal normalization by inhibiting the crosstalk between cancer cells and pancreatic stellate cells, increasing the penetration and anti-tumor efficacy of nanopolyplex.	118
	SB-431542	β-cyclodextrin-conjugated heparin and pH-responsive pseudorotaxane for codelivery of DOX and SB431542	4T1	Female BALB/c mice	Dual α-amylase/pH responsive NPs modulate the TME and subsequently amplify the therapeutic effect of DOX.	119
	LY2157299	Anti-PD-1 and LY2157299-loaded gelatinase-responsive mPEG-peptide -PCL NPs	H1299	C57BL/6 mice	Gelatinase-responsive NPs overcome the immunotherapeutic resistance and achieve superior anti-tumor efficacy by cosuppressing PD-1 and TGF-β.	132
ROS stimuli	LY2109761	HCD and FC conjugate shells with BzPGA cores for codelivery of LY210976 and Ce6	4T1	Female BALB/c mice	ROS-responsive core-shell NPs release the LY2109761 in a coordinated manner to achieve timely suppression upon PDT.	120
	siTGF-β	10-Hydroxycamptothecin grafted polymer core and PEG-PLL-DMMA shell loaded with siTGF-β	B16F10	C57BL/6 female mice	The pH/ROS-responsive size-loss and charge-reversal nanoplatform improves chemoimmunotherapy by reprogramming the TME.	122
pH stimuli	LY2157299	PEG-PDPA copolymer self-assembled with Ce6 and LY2157299	CT26	Male BALB/c mice	The combination of nanoplatform-mediated SDT and TGF-β/SMAD blockade enhances the therapeutic efficacy of anti-PD-L1.	123
	LY2157299	Polymeric clustered NPs composed of PEG-PCL, PCL and PCL-CDM-PAMAM for codelivery of LY2157299 and siRNA silencing PD-L1	Panc02	C57BL/6 mice	Polymeric clustered NPs reverse the immunosuppressive microenvironment and activate the PD-1/PD-L1 checkpoint for enhanced immunotherapy.	124
	Ingenol-3-mebutate	Single alcoholic hydroxyl (-CH(CH_3_)-OH polymeric vesicles loaded with ingenol-3-mebutate	Sarcoma-180	Male C57BL/6 mice	These pH-sensitive polymeric vesicles overcome the hydrophobicity and pH instability of ingenol-3-mebutate, leading to the activation of anti-tumor immunity.	125
	LY2157299	Nanomicelles composed of PgA-PAA conjugation for delivery of LY2157299	HLF	-	These nanomicelles ensure the stability of LY2157299 in acidic pH of gastrointestinal tract.	126
Hypoxic stimuli	LY2157299	Amphiphilic polymer modified with enamine N-oxides and CBP peptide for delivery of LY2157299 and GEM prodrug	KPC, NIH3T3	Male C57BL/6 mice	These charge-reversal NPs can initiate transcytosis across the ECM barrier to achieve deep penetration in PDAC.	127
Polymer based hydrogels and nanogels	LY3200882	Thermosensitive hydrogel incorporating regorafenib and ROS-responsive nanogel loaded with LY3200882	CT26	Female C57BL/6 mice	This integrated hydrogel/nanogel system not only restrains the potential tumor metastatic threats induced by regorafenib, but also activates the immune responses by TGF-β blockade.	128
Gene medicine-loaded polymeric NPs	SB-505124	β-cyclodextrin-PEI loaded with SB-505124 and the gene encoding murine IL-12	B16, A549	Female C57BL/6 mice	This codelivery system facilitates the sustained release of the SB-505124, ensuring efficient transduction of the adenoviral vector encoding the IL-12 gene.	129
	siTGF-β	PLA-based nanovaccine for codelivery of α-Lactalbumin antigens, Toll like receptor ligands, and glutamate chitosan/siTGF-β	4T1, EO771	Female BALB/c mice, Female C57BL/6 mice	The synergistic combination of nanovaccine and OX40 suppresses tumor progression by eliciting antigen-specific immunity and silencing TGF-β.	130
	siTGF-β	Polypeptide loaded with TGF-β and Cox2 siRNA	Hepa1-6	Female C57BL/6 mice	Gene silencing of TGF-β and Cox2 induces the activation of the immune TME by enhancing T cell penetration.	133
Active targeted polymeric NPs	SD-208	Anti-CD8a F(ab')_2_ fragments conjugated PEG-PLGA polymeric NPs	MC38, B16	C57BL/6 mice	These T cell-targeting NPs restore the function of effector T cell and other suppressed immune cells and inhibit the tumor growth.	131

HES: hydroxyethyl starch; PLA: polylactide; DOX: doxorubicin; HCD: hyaluronic acid-aldehyde-monosubstituted β-cyclodextrin; FC: hexadecanol-conjugated ferrocene; BzPGA: benzyl-modified poly(γ-glutamic acid); PgA: poly(glycolic acid); PEI: polyethyleneimine; PGA: polygalacturonic acid; PAA: polyacrylic acid; PEG-PLL-DMMA: polyanion poly(ethylene glycol)-blocked-poly(L-lysine)-modified dimethylmaleic anhydride; PDPA: Poly(2-(diisopropylamino)ethyl methacrylate); PCL: poly(ε-caprolactone); PCL-CDM-PAMAM: poly(amidoamine)-graftpolycaprolactone; GEM: gemcitabine; PEG-PAsp: poly(ethylene glycol)-poly(aspartic acid) block copolymer; SDT: sonodynamic therapy; ROS: reactive oxygen species.

**Table 3:** Summary of inorganic NPs targeting TGF-β.

Characteristic	Nanoparticle/carriers	Cancer cell type	Model	Outcome	Ref.
Inorganic NPs suppress the TGF-β signaling pathway	AuNPs	MBT-2	Female (either C3H/HeN or NOD-SCID) mice	AuNPs attenuate the EMT through the formation of AuNP-TGF-β1 conjugates.	136
	AuNPs	A2780-CP20, CAFs	-	AuNPs with size in 20 nm inactivate the CAFs by blockade of TGF-β1, PDGF, and other markers of CAFs.	137
	Carbon nanodiamonds	A549	Nude mice	Nanodiamonds suppress the TGF-β by inducing the lysosomal decomposition of TGF-β receptors.	138
	Metallofullerenol-based Gd@C_82_(OH)_22_ NPs	MDA-MB-231	Female BALB/c nude mice	Gd@C_82_(OH)_22_ NPs eliminate the CSCs and impede the EMT via suppression of HIF-1α and TGF-β.	139
	TiO_2_ NPs	A549	Zebrafish, C57BL/6 mice	TiO_2_ NPs suppress the EMT progression by binding to the TβRI/II.	140
Inorganic NPs activate the TGF-β signaling pathway	Nano-TiO_2_	SW480	-	Nano-TiO_2_ exhibits the toxic and side effect by activation of the TGF-β/MAPK and Wnt pathways.	142
	Nano NiO	HepG2	Male wistar rats	Nano NiO promotes the EMT progression and ECM deposition through TGF-β signaling pathway, resulting in hepatic fibrosis.	143
	TiO_2_ nanofibers	A549	BALB/c nude mice	TiO_2_ nanofibers cause lung epithelial cells to obtain an aggressive tumor phenotype.	144
	PEI coated MNPs	HeLa	-	PEI-MNPs lead to tumor progression through the induction of autophagy and the activation of the NF-κB and TGF-β.	145
	Graphene oxide	4T1	Female BALB/c mice	GO activates the TGF-β signaling to exert prometastatic effects in a dose-dependent manner.	146
	AgNPs	-	Male and female ICR mice	Small-sized AgNPs are more active at triggering toxicological or biological responses than large-sized AgNPs.	147
	AlO NPs	KLN 205, HeLa, A549, SKOV3	Female DBA/2 mice	Wire shaped AlO, but not spherical shaped AlO NPs, leads to tumor-associated inflammation and metastasis.	148

AuNPs: gold NPs; TiO_2_: titanium dioxide NPs; NiO NPs: nickel oxide NPs; PEI: polyethyleneimine; MNPs: magnetic NPs; GO: graphene oxide; AlO NPs: aluminum oxide NPs; AgNPs: silver NPs; TGF-β: transforming growth factor-β; CAFs: cancer-associated fibroblasts; EMT: epithelial-mesenchymal transition; ECM: extracellular matrix.

**Table 4:** Summary of hybrid NPs, lipid-polymeric NPs and small-molecule NPs targeting TGF-β for cancer treatment.

Characteristic	TGF-β pathway antagonists	Nanoparticle/carriers	Cancer cell type	Model	Outcome	Ref.
Hybrid NPs	RLX	RLX-SPION	PANC-1	Male CB17 SCID mice	RLX-2 conjugated SPIONs enhance the chemotherapeutic efficacy of GEM by inhibiting the differentiation of PSCs into CAF-like myofibroblasts.	149
	Anti-TGF-β antibody	Anti-TGF-β and RGD2 conjugated Fe_3_O_4_/Gd_2_O_3_ hybrid NPs for delivery of sorafenib	MC38	C57BL/6 mice	This MRI-guided nanosystem achieves synergistic effect of ferroptosis and TGF-β blockade.	150
	siTGF-β	Angiopep LipoPCB (Temozolomide+BAP/siTGF-β)	GL261	C57BL/6 mice	Traceable NPs efficiently cross the blood-brain barrier, enabling combined immunotherapy and chemotherapy for intracranial glioblastoma.	151
	siTGF-β	PEI-PEG nanogels loaded with TGF-β siRNA and Fe_3_O_4_ NPs	S180	Male BALB/c mice	This nanosystem loaded with TGF-β1 siRNA performs MR imaging-guided gene therapy of sarcoma.	164
	siTGF-β1	Cysteamine-functionalized AuNPs for delivery of siTGF-β1	HepG2	Female athymic nude mice	These AuNP-siRNA nanocomposites deliver siTGF-β1 into implanted tumor cells and protect it from physiological conditions.	152
	SB525334	Anti-CD8 VHH nanobody conjugated Amph-NPs for delivery of SB525334	Lymphocytes	C57BL/6 mice	The targeted nanosystem promotes the delivery of the SB525334 to T cells, while free SB525334 does not affect T cell functionality.	153
	Maclura tricuspidata extract	Au NPs-conjugated maclura tricuspidata extract (MT-GNPs)	HepG2, SK-Hep-1	-	MT-GNPs reverse the EMT by TGF-β1 blockade.	165
	-	cRGD-targeted Au NPs loaded with the gefitinib and IR780	PC-9GR	BALB/c nude mice	Combination of SDT and low-temperature PTT mediated by hybrid Au NPs overcomes the EGFR-TKI resistance by inactivation of TGF-β/PDLIM5/SMAD resistance pathway.	166
	LY2157299	Anti-L1CAM antibody functionalized hybrid NPs intergrating gelatin, protein A, diatomite and Au NPs	SW620	Athymic nude mice	These hybrid nanosystems achieve targeted precise therapy for treatment of metastatic colorectal cancer.	167
	Anti-TGF-β antibody	CuS NPs modified with PEG-anti-TGF-β for delivery of ATM inhibitor	HepG2, H22	Female BALB/c mice	Hybrid CuS NPs modified with anti-TGF-β antibody achieve ATM inhibitor-mediated chemotherapy and CuS-mediated low-temperature PTT with amplified specificity.	154
	-	Cu_2_(OH)PO_4_ NPs coated with PAA	U87MG, U251 and T98G	BALB/c nude mice	The PTT mediated by Cu₂(OH)PO₄ is related to MSH6-CXCR4-TGF-β1 axis.	155
	SB525334	GSH-responsive MSN	Panc02	C57BL/6 mice	Modulating neutrophil polarization via nanomedicine facilitates irreversible electroporation and anti-PD-1-mediated immune checkpoint blockade.	156
	siTGF-β	PEI-coated MSN for delivery of PD-L1 inhibitor JQ1 and siTGF-β	A375, T lymphocyte cell	-	Hybrid siRNA NPs regulate the TME by protecting tumor escape from immune system.	168
	Halofuginone	Mesoporous platinum NPs loaded with halofuginone	4T1	Female BALB/c nude mice	The halofuginone-loaded theranostic mesoporous platinum nanosystem modulates the ECM with no observed systemic toxicity.	169
	SB-505124	SB-505124-loaded PEGylated MSN intergrated with Mn^2+^ and Fe^2+^	CT26	C57BL/6 mice	Multimetal nanozymes remodel the TME for improving the efficacy of catalytic tumor therapy.	157
	Curcumin	CaCO_3_/PDA NPs loaded with curcumin and ropivacaine	4T1	BALB/c mice	Blockade of TGF-β inhibits the expression of TRPV1, achieving comfortable immunotherapy with palliative hyperalgesia.	170
Lipid-polymeric NPs	SB505124	Liposomal polymeric gels	-	Female B6 albinomice	Hybrid core-shell nanoplatform enhances tumor immunotherapy against melanomas through the activation of immune responses.	158
	Esomeprazole	Lipid NPs loaded with mRNA and esomeprazole	HeLa, L929 and B16-OVA	Female C57BL/6 mice	Lipid formulations loaded with esomeprazole promote the escape of the mRNA from the endo-/lysosomes and deep penetration of lipid NPs.	159
	Vactosertib	Vactosertib-loaded liposomes delivering paclitaxel-loaded PEG-PLGA nanospheres	Panc-1	BALB/c nude mice	The size switchable nanoplatform achieves the sequential cascade release of drugs for enhanced chemotherapy of stroma-rich tumors.	160
	SB-505124	Nanoliposome loaded with ICG, SB-505124, and zoledronic acid	4T1	Female BALB/c mice	The nanosystem modulates the immunosuppressive microenvironment by combination of photothermal immunotherapy and TGF-β blockade.	171
	siTGF-β1	Hybrid NPs composed of PLGA, DSPE-mPEG, and cRGD-conjugated DSPE-PEG	4T1	Female BALB/c mice	The nanoplatform remodels the suppressive immune TME to boost the immunotherapy.	172
	siTGF-β1	Lipid- PEG NPs	A549, T cells	Female BALB/c nude mice	The siRNA Lipid NPs efficiently defeat paclitaxel-resistant lung cancer.	173
Small- molecule NPs	OA	OA NPs and nanogels	4T1	Female BALB/c mice	OA nanogels exhibit the highest tumor inhibition efficiency on α-SMA among the different OA formulations.	161
	OA	OA nanogel comprising rose bengal and methylene blue	4T1	Female BALB/c mice	Tri-component programmable nanogel inactivate the CAFs via TGF-β blockade for PTT/SDT-assisted immune therapy.	162
	SB505124	Self-assembled bioregulators incorporating Ce6, SB505124 and lonidamine	CT26	BALB/c mice	The combination of PDT and reversal of the immunosuppressive TME synergistically amplify the immunotherapy for treatment of colorectal cancer.	163

RLX: human relaxin-2; SPION: superparamagnetic iron nanocubes; PSC: pancreatic stellate cells; GEM: gemcitabine; RGD2: arginine-glycine-aspartic dimer; siTGF-β1: TGF-β1 siRNA; MRI: magnetic resonance imaging; ATM: ataxia telangiectasia mutated; PEI: polyethyleneimine; PEG: poly(ethylene glycol); PAA: polyacrylic acid; OA: oleanolic acid; MSN: mesoporous silica NPs; PLGA: poly(lactic-co-glycolic acid); ICG: indocyanine green; Ce6: Chlorin e6; TGF-β: transforming growth factor-β; CAFs: cancer-associated fibroblasts; EMT: epithelial-mesenchymal transition; ECM: extracellular matrix; PTT: photothermal therapy; SDT: sonodynamic therapy.

**Table 5:** Summary of biomimetic NPs, immune cell therapy and protein-based NPs targeting TGF-β for cancer treatment.

Characteristic	TGF-β pathway antagonists	Nanoparticle/carriers	Cancer cell type	Model	Outcome	Ref.
Cell membrane coated biomimetic NPs	SD-208	Macrophage membrane-coated NPs loaded with SD-208	4T1	Female BALB/c mice	Macrophage membrane-coated NPs effectively suppress tumor metastasis and reprogramed the immunosuppressive TME by blocking M2-type macrophage differentiation.	174
	LY2157299	Macrophage membrane and conjugates of anti-PDL1 and PEGylated phospholipids as shell, gold nanocages loading LY2157299 as core	CT26	BALB/c mice	Nanocomposites exert a promising suppression on both primary and metastatic tumor progression by dual suppression of PD-1/PD-L1 and TGF-β.	175
	SB-505124 hydrochloride	Fe_3_O_4_ nanoclusters coated with anti-PD-1 antibody conjugated leukocyte membrane for delivery of SB-505124 hydrochloride	4T1, B16F10	Female BALB/c mice, C57BL/6 mice	Combination strategy of immunomodulation and ferroptosis shows superior therapeutic performance.	176
	SB525334	Autologous neoantigens-primed dendritic cell membranes with liposomes for delivery of SB525334	B16-OVA	C57BL/6 mice	The dual blockade of PD-1 and TGF-β by bionic NPs reshapes the TME and enhances CD8^+^ T cell infiltration.	177
	T cell membranes with high expression of TβR	T cell membrane coated CaO_2_ NPs	B16-F10	Female C57BL/6	The T cell-mimicking Ca^2+^ nanogenerator provides diverse benefits for cytotoxic lymphocyte cell-mediated chemoimmunotherapy.	178
	LY2157299	Cancer cell membrane- coated trimetallic nanozyme	LA795	Male BALB/c mice	Combination of polarization of macrophages by TGF-βi and nanozyme achieves superior therapeutic effects under laser irradiation.	179
	SB525334	*S. aureus* membrane-coated nanosystem with a core of lanthanidedoped scintillators and a hollow macroporous silica shell to entrap SB525334 and Roussin's black salt	CT 26, B16F10, B16F10-OVA	Female BALB/c mice, C57BL/6 mice	The generation of ONOO^-^ works synergistically with GSH-responsive released SB525334, inducing tumor-associated neutrophils polarization toward the anti-tumor N1 phenotype.	180
Immune cell therapy	LY2157299	HES-PCL NPs loaded with LY2157299 and ICG	Raji	BALB/c female mice	The synergistic combination of the nanosystem and CAR-T cells demonstrates higher antitumor activity than CAR-T cell monotherapy.	181
	SB505124	Nanoemulsion loaded with SB505124 and selenocysteine	MDA-MB-231	Female nude mice	The pretreatment of nanoemulsion enhances the killing potency and accumulation of NKs for enhanced NK cell adaptive therapy.	182
	LY2157299	Chemically prime NK cells by LY2157299- loaded polymeric nanogels	PC-3	BALB/c nude mice	Reversal of immunosuppressive TME through NK cell-priming activity and blockade of TGF-β performs effective anti-tumor response.	183
	SB525334	Anti-Thy1.1 targeted PEGylated liposomes, anti-CD45 targeted PEGylated liposomes	B16F10	C57BL/6 mice	Two liposomes with two different receptors were developed to deliver SB525334 to T cells in adoptive cell therapy.	184
Protein-based NPs	-	Unfolded HAS coated perfluorotributylamine NPs	CT26	BALB/c mice	The unfolded HSA-coated NPs inhibit the platelet-derived TGF-β, exerting inhibitory effects on tumor metastasis and NK cells immune surveillance.	185
	Tamoxifen	Heptamethine cyanine IR68-tamoxifen conjugate coated albumin	4T1	Female BALB/c mice	Tumor-targeting NPs boost radiotherapy by reversing tumor hypoxia as well as by inhibiting PD-L1 and TGF-β.	187
	Tamoxifen	Albumin coated heptamethine cyanine MHI-tamoxifen conjugate	4T1	Female BALB/c mice	Mitochondrial targeted nanosystem through dual inhibition of PD-L1and TGF-β reverses the resistance of photodynamic immunotherapy.	188
	LY2157299	Albumin modified by brain-targeting peptide for delivery of LY2157299 and celastrol	GL261	-	The biomimetic NPs achieve efficient anti-tumor effects against glioma through suppressing the proportion of M2 TAMs and TGF-β1.	189

PEG: poly(ethylene glycol); PD-1: programmed cell death protein 1; PD-L1: programmed cell death ligand 1; ONOO^-^: peroxynitrite; HES: hydroxyethyl starch; PCL: poly(ε-caprolactone); ICG: indocyanine green; CAR-T: chimeric antigen receptor T cell; TME: tumor microenvironment; NK cells: natural killer cell; HAS: human serum albumin.
